# Robustness of the Dorsal morphogen gradient with respect to morphogen dosage

**DOI:** 10.1371/journal.pcbi.1007750

**Published:** 2020-04-06

**Authors:** Hadel Al Asafen, Prasad U. Bandodkar, Sophia Carrell-Noel, Allison E. Schloop, Jeramey Friedman, Gregory T. Reeves

**Affiliations:** 1 Department of Chemical and Biomolecular Engineering, North Carolina State University, Raleigh, North Carolina, United States of America; 2 Genetics Program, North Carolina State University, Raleigh, North Carolina, United States of America; Northeastern University, UNITED STATES

## Abstract

In multicellular organisms, the timing and placement of gene expression in a developing tissue assigns the fate of each cell in the embryo in order for a uniform field of cells to differentiate into a reproducible pattern of organs and tissues. This positional information is often achieved through the action of spatial gradients of morphogens. Spatial patterns of gene expression are paradoxically robust to variations in morphogen dosage, given that, by definition, gene expression must be sensitive to morphogen concentration. In this work we investigate the robustness of the Dorsal/NF-κB signaling module with respect to perturbations to the dosage of maternally-expressed *dorsal* mRNA. The Dorsal morphogen gradient patterns the dorsal-ventral axis of the early *Drosophila* embryo, and we found that an empirical description of the Dorsal gradient is highly sensitive to maternal *dorsal* dosage. In contrast, we found experimentally that gene expression patterns are highly robust. Although the components of this signaling module have been characterized in detail, how their function is integrated to produce robust gene expression patterns to variations in the *dorsal* maternal dosage is still unclear. Therefore, we analyzed a mechanistic model of the Dorsal signaling module and found that Cactus, a cytoplasmic inhibitor for Dorsal, must be present in the nucleus for the system to be robust. Furthermore, active Toll, the receptor that dissociates Cactus from Dorsal, must be saturated. Finally, the vast majority of robust descriptions of the system require facilitated diffusion of Dorsal by Cactus. Each of these three recently-discovered mechanisms of the Dorsal module are critical for robustness. These mechanisms synergistically contribute to changing the amplitude and shape of the active Dorsal gradient, which is required for robust gene expression. Our work highlights the need for quantitative understanding of biophysical mechanisms of morphogen gradients in order to understand emergent phenotypes, such as robustness.

## Author summary

The early stages of development of an embryo are crucial for laying the foundation of the body plan. The blueprint of this plan is encoded in long-range spatial protein gradients called morphogens. This positional information is then interpreted by nuclei that begin to differentiate by expressing different genes. In fruit fly embryos, the Dorsal morphogen forms a gradient along the dorsal-ventral axis, with a maximum at the ventral midline. This gradient, and the resulting gene expression patterns are extraordinarily robust to variations in developmental conditions, even during early stages of development. Since positional information is interpreted in terms of concentration of the morphogen, one would expect that doubling or halving dosage would result in disastrous consequences for the embryo. However, we observed that development remains robust. We quantified the effect of dosage by experimentally measuring the boundaries of 2 genes,—*sna* and *sog*, expressed along the DV axis and found that variation in the boundaries of these genes was minimal, across embryos with different dosages of Dl. We then used a mathematical model to discern components of the Dl system responsible for buffering the effects of dosage and found three specific mechanisms–deconvolution, Toll saturation and shuttling. These biophysical mechanisms, built into the early developing embryo, ensure robustness of target gene expression when dosage of the Dl morphogen is altered.

## Introduction

The morphogen concept forms the basis of many models of developing tissues. Through their concentration gradients in space, morphogens send positional information to cells and direct them to develop in specific ways depending on their location within a tissue. The roles of these signals range from the development of the initial polarities of embryos to specification of cell identity in specific tissues, and the nervous system in both vertebrates and *Drosophila* [1]. Tissue patterning is often initiated by the cells’ concentration-dependent response to the morphogen gradient: cells throughout the tissue are subject to different concentrations of morphogen, depending on their position within the field, and accordingly, express distinct target genes. Thus, the quantitative shape of the morphogen gradient is critical for patterning, with cell-fate boundaries established at specific concentration thresholds. The cells’ sensitivity to morphogen concentration also implies that any shift in the morphogen distribution is expected to result in an accompanying shift in patterning. Therefore, perturbations to the morphogen dosage or production rate, which should change the morphogen distribution, should in turn perturb gene expression patterns.

Indeed, early models of morphogen gradient formation assumed the gradient scaled globally with the morphogen dose (*e*.*g*., when one copy of the gene encoding the morphogen is lost, the entire distribution is divided by two). Such “dosage-scaling” models predicted that catastrophic shifts in target gene expression domains would occur when the dose of morphogen is altered [2,3]. In contrast, experimental observations have shown that the spatial positioning of morphogen target genes shift only minimally when morphogen dosage is perturbed [2–5], with some notable exceptions, such as Dpp-dependent patterning in the early embryo [6,7]. Thus, there exists a paradox between the sensitivity of cells to morphogen concentration and the robustness of tissue patterns with respect to morphogen dose, which implies a mechanism that prevents robust morphogen gradient systems from scaling with morphogen dose. One such mechanism is self-enhanced ligand degradation, where the ligand (morphogen) upregulates its own inhibitor, and which has been suggested to explain experimentally-observed robustness [3,4,8,9]. However, this mechanism does not apply to all morphogen gradient systems. In particular, the self-enhanced degradation mechanism has not been observed in the Dorsal/NF-κB signaling network in *Drosophila* embryos.

**Fig 1:**
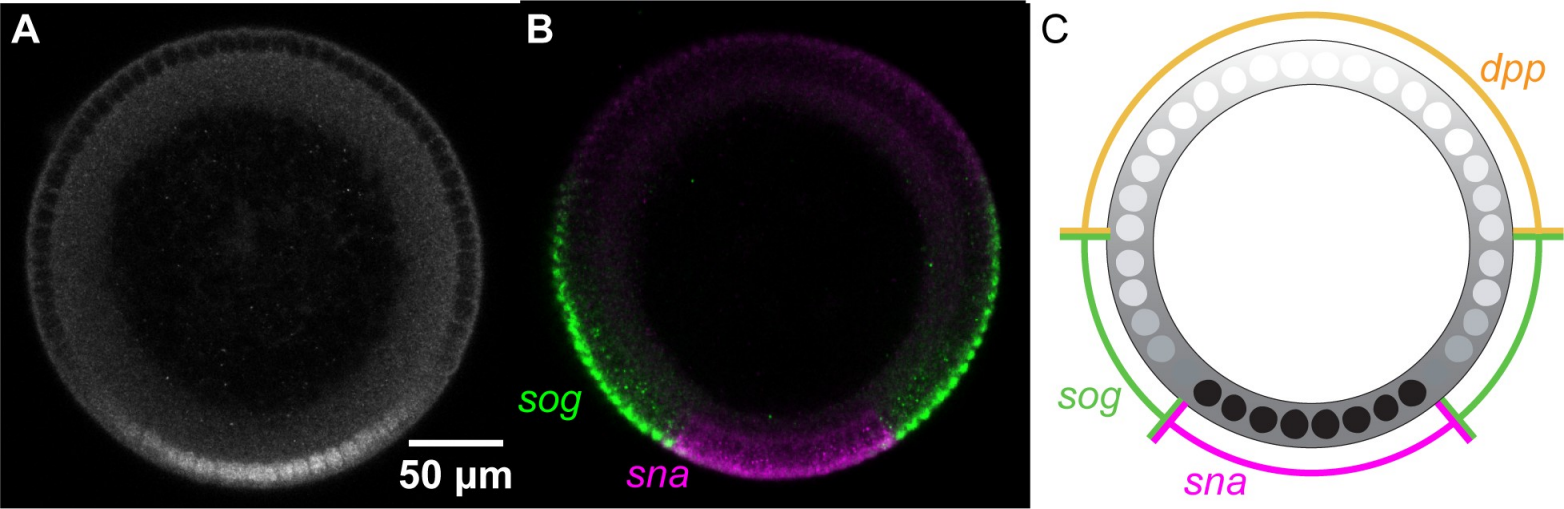
The protein Dorsal patterns the DV axis of the *Drosophila* embryo. (A) An antibody staining against Dorsal in an NC 14 embryo. (B) mRNA expression of a variety of the Dorsal target genes *sna* and *sog*. (C) Illustration of the borders of gene expression. We use these borders to quantify and compare the extent of domain of Dl target genes. Embryo cross-sections are oriented so that ventral is down.

The NF-κB module, conserved from flies to humans, is implicated in several cellular responses/phenotypes, including tissue patterning, inflammation, innate immunity, proliferation/apoptosis, and cancer [10–14]. The maternal transcription factor Dorsal (Dl), homologous to mammalian NF-κB, patterns the dorsal-ventral (DV) axis of the developing *Drosophila melanogaster* embryo to specify mesoderm, neurogenic ectoderm, and dorsal ectoderm cell fates reviewed in [15–18]. In the early embryo, Dl protein is initially uniformly distributed around the DV axis. During nuclear cleavage cycle (nc) 10, the nuclei migrate to the periphery of the syncytial blastoderm and the Dl gradient begins to be established. The IκB homolog Cactus (Cact), which is also maternally-supplied, binds to Dorsal, retaining it outside the nuclei. Toll, the *Drosophila* homolog of the Interleukin 1 receptor, is active on the ventral side of the embryo, where it signals through Pelle kinase to phosphorylate the Dl/Cact complex [19], which results in dissociation of Dl from Cact, allowing Dl to enter the nuclei, where it regulates gene expression. Because Toll signaling is spatially asymmetric, a nuclear gradient of Dl forms, with a peak at the ventral midline and a Gaussian-like decay in space to become nearly flat at approximately 45% of the embryo’s circumference (**Fig 1A**) [5,20]. From 45% to 100% ventral-to-dorsal coordinate, the gradient has a shallow downward slope to achieve non-zero basal levels at the dorsal midline [5,20,21]. Our computational studies have suggested the non-zero basal levels are primarily composed of Dl/Cact complex in the dorsal-most nuclei, not free Dl [22].

As shown in **Fig 1B**, different genes are turned on at different concentrations of Dl [16,23]. It can be both an activator and a repressor of transcription. At high concentrations of Dl on the ventral side of the embryo, high threshold genes such as *snail* (*sna*) are expressed. In the lateral part of the embryo, intermediate Dl levels activate the expression of low threshold genes such as *short gastrulation* (*sog*). The domains of these genes can be quantified using measurements of the dorsal border and ventral border (**Fig 1C**). The expression of *sna* is instrumental in establishing the boundary between the mesoderm and the neuroectoderm at ~20% DV length. Similarly, the expression of other Dl targets such as *sog* and *dpp* help establish the boundary between the neuroectoderm and the dorsal ectoderm. If the expression boundaries of Dl targets that help pattern the embryo in the DV axis is altered, it may result in significant changes to the body plan which could make the embryos inviable.

While the copy number of maternal *dl* has been shown to affect the Dl gradient and downstream tissue structure, the phenotypes are subtle. Embryos from mothers heterozygous for a *dl* null allele (1x *dl*) have shorter and flatter Dl gradients as compared to wildtype [5,24–26]. Furthermore, they appear wider when normalized Dl gradients are compared between 1x *dl* and wildtype. While these embryos have a weakly dorsalized phenotype, female flies with a half dose of *dl* produce a high fraction of viable progeny at room temperature [27,28]. Furthermore, measurements in a handful of embryos (n < 12) found no statistical shift in the *sog* expression domain [5], and a shift of roughly only one cell diameter in the *sna* domain [26]. The altered shape of the Dl gradient has recently been attributed to a combination of two novel observations. First, active Toll receptor complexes are saturated by Dl/Cact complex [26]. And second, Cact acts to facilitate the diffusion of Dl (i.e., “shuttling” of Dl by Cact), which results in a net flux of Dl to the ventral side of the embryo [26]. Together, these processes act to accumulate Dl on the ventral side in wildtype embryos, but accumulate Dl in ventral-lateral regions in 1x *dl* embryos. Furthermore, experimental evidence strongly suggests the shuttling mechanism is required for viability of 1x *dl* embryos, as embryos from heterozygous *dl* mothers that also have compromised shuttling are non-viable [26].

In a similar manner, embryos with overexpression of excess, transgenic copies of *dl* (4x *dl*) are only weakly ventralized, and a large fraction still hatch [29]. Given the subtlety of the 1x and 4x *dl* phenotypes, and the viability of the embryos, one may ask whether this implies the Dl gradient system is robust, and if so, whether the robustness requires special mechanisms, such as shuttling and Toll saturation [26]. As mentioned above, dosage-scaling models are typically sensitive to dosage. However, a dosage-scaling model of the Dl gradient has not been analyzed for robustness of gene expression with respect to variations in morphogen dosage.

In this work, we used empirical and computational modeling, together with quantitative measurements of the Dl gradient and domains of target gene expression, to investigate the robustness of the Dl gradient system with respect to dosage of maternal *dl*. First, we showed that a dosage-scaling formulation of the Dl gradient has a high sensitivity to the maternal dosage of *dl*, even in the best-case scenario, in which basal levels are composed primarily of Dl/Cact complex and there is negligible Dl activity at the dorsal midline [22]. In particular, in the absence of a mechanism to prevent dosage-scaling, doubling or halving the maternal *dl* dosage is predicted to result in drastic perturbations to gene expression. Next, we experimentally measured gene expression domains and the Dl gradient width in embryos from mothers with *dl* dosages of 1x (heterozygous null for maternal *dl*), 2x (wildtype), and 4x (expressing two copies of a *dl* rescue construct; Carrell et al., 2017; Reeves et al., 2012) and showed that, in contrast to the predictions of the dosage-scaling model, the perturbations to patterns are minimal. To identify the possible mechanism for this robustness, we analyzed a computational model of the Dl/Cact system. Our model is based on previously published models in which Dl and Cact can interact, enter the nuclei, and diffuse between “cytoplasmic compartments” surrounding the nuclei [21,22,24,26,30]. The active Toll signaling complex, which is limited to the ventral side of the embryo, acts as a Michaelis-Menten-like enzyme to favor dissociation of the Dl/Cact complex.

We conducted a random search over six orders of magnitude for all free parameters of the model and filtered the robust parameter sets by constraining the results using our measurements of Dl target gene expression in 1x, 2x, and 4x embryos (see Methods). Of ~200 000 parameter sets explored, about 1150 parameter sets were deemed robust. Our analysis of the robust parameter sets showed that robustness can rarely be achieved unless (1) the free Dl nuclear levels drop to near zero on the dorsal side of the embryo [22], (2) significant facilitated diffusion by Cact occurs [26], and (3) active Toll signaling can be saturated by Dl/Cact complex [26]. Furthermore, the robust parameter sets demonstrated a bias in the amplitudes of the 1x and 4x embryos, relative to the 2x embryos. In particular, simulations of robust 1x embryos had Dl gradient amplitudes between 50% and 100% of the 2x embryos’ amplitudes, while the 4x Dl gradient amplitudes were between 100% and 150% of the 2x embryos’. We performed live imaging of 1x, 2x, and 4x *dl-gfp* embryos, and experimentally found amplitude ratios were largely consistent with the computational results.

Quantitative analysis can be used to assess rigorously the robustness of different patterning models. Applying similar modelling approaches to other systems might identify additional mechanisms that underlie robust patterning by morphogen gradients in development.

## Results

### Sensitivity of a dosage-scaling model of the Dl gradient

Early models of morphogen gradients exhibited “dosage-scaling,” in that these descriptions of the morphogen gradient scaled globally, in a multiplicative manner, with morphogen dosage. Morphogen gradients predicted by these models were highly sensitive with respect to morphogen dosage [3,31,32]. However, these models focused on exponential-like morphogen distributions, whereas the Dl gradient is Gaussian-shaped [5,20,26]. Therefore, to determine the extent to which the robustness of the Dl system may be inherent to the Gaussian shape of the Dl gradient, versus how much of the robustness requires a special mechanism, we analyzed an empirical, dosage-scaling description of the gradient.

Let *c*(*x*) be the dimensionless distribution of nuclear Dl as a function of the relative DV coordinate *x*:

$$c(x)=\alpha \left[\exp\left(-\frac{x^{2}}{2\sigma^{2}}\right)+m|x|+b\right],$$ (1)

where *α* is a proportionality constant related to morphogen dosage, *σ* represents the spatial width of the Dl gradient, *m* is the shallow, downward slope of the Dl gradient tail, and *b* represents the basal levels of the gradient, related to the levels of Dl that is present in the dorsal-most nuclei. From empirical measurements, *b*≈0.4 and *m*≈−0.1 [20].

**Fig 2:**
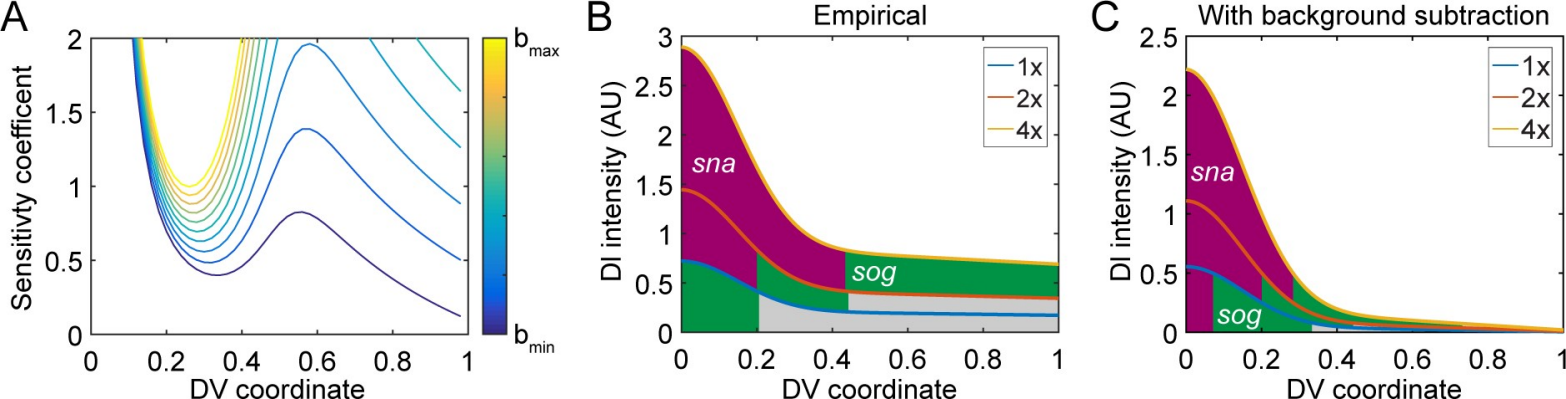
Theoretical consideration of the sensitivity coefficient. (A) Testing whether a lower value of the parameter b could result in a lower sensitivity. (B) The empirical prediction shows that 1x embryos completely lose *sna* expression, while 4x embryos have an overexpanded domain of *sna*, and lose *dpp* completely. (C) The prediction when lower b values were used.

To calculate the robustness of the predicted gene expression boundaries with respect to changes in *α*, we performed a sensitivity analysis. Let the sensitivity coefficient of a gene expression border with respect to maternal *dl* dosage be defined as *ϕ*≡(∂ ln *x*_*g*_/∂ ln *α*)_*θ*_, where *x*_*g*_ is the location of gene expression boundary and *θ* is the threshold in Dl nuclear concentration required to express the gene (see Materials and Methods for more information). We found the model of the Dl gradient described by Eq (1) has sensitivity coefficients of one or greater (Fig 2A). As a loose rule of thumb in engineering circles, it is desirable to have sensitivity coefficients to be 0.3 or less [32,33]; thus, the gradient described by Eq (1) is highly sensitive.

Previously, it was found that a model in which both Dl and Dl/Cact complex are present in the nucleus was more consistent with experimental results than one in which only free Dl is allowed to enter the nucleus [22]. This model was also more robust to noise in Dl levels, as removing, or “deconvolving” the Dl/Cact contribution from the fluorescence measurements reduces the free, active Dl nuclear concentration to near zero at the dorsal midline [22]. Therefore, we asked whether empirically modeling the presence of Dl/Cact complex in the nuclei could also improve the predicted robustness with respect to maternal *dl* dosage. In this case, Eq (1) represents the sum of the two Dl-containing species. In this paper, we define deconvolution as the act of computationally separating the Dl/Cact nuclear concentration from the sum (Eq (1)) to result in the active, “true” Dl gradient (i.e., free Dl). Deconvolution is required if Dl/Cact complex is appreciably present in the nucleus, and has the implication that free Dl nuclear concentration is low, perhaps near zero, at the dorsal midline. Our previous work has suggested the Dl/Cact contribution is roughly constant across the DV axis [22], so that empirically, the active Dl gradient can be modeled by Eq (1) with a much lower value of *b*. If we set *b* = 0.11, so that the intensity of free nuclear Dl in the dorsal-most nuclei is 1% of the intensity in the ventral-most nuclei, the sensitivity of gene expression is improved markedly (Fig 2A). However, even in this best-case scenario, the minimum sensitivity coefficient (located at *x*_*g*_ = 0.34) is roughly 0.4, which is higher than the suggested rule-of-thumb of 0.3. Gene expression boundaries located elsewhere experience even higher sensitivities.

To put the problem in more experimentally concrete terms, we can use Eq (1) to predict the outcome of deleting one copy of maternal *dl* (1x *dl*), or expressing two extra copies (4x *dl*). Let *α* = 1 to represent the wildtype dosage of maternal *dl*, so that *α* = 0.5 and *α* = 2 represent the 1x and 4x embryos, respectively. In the perturbed cases, the predicted DV gene expression profile in the embryo would result in lethality: 1x embryos completely lose *sna* expression, while 4x embryos have a highly expanded domain of *sna* and lose *dpp* completely (Fig 2B). As with the sensitivity coefficient above, if *b* is lowered, the effects on gene expression are less severe (Fig 2C). However, the empirical model still predicts lethality: 1x embryos express *sna* in < 10% of the DV axis [26], and 4x embryos have severely reduced *dpp* expression. We conclude that robustness does not arise simply from a Gaussian shape in a dosage-scaling context, and thus, there must be a mechanism by which the embryo compensates for changes in the maternal *dl* dosage.

### Robustness of Dl-dependent gene expression

**Fig 3:**
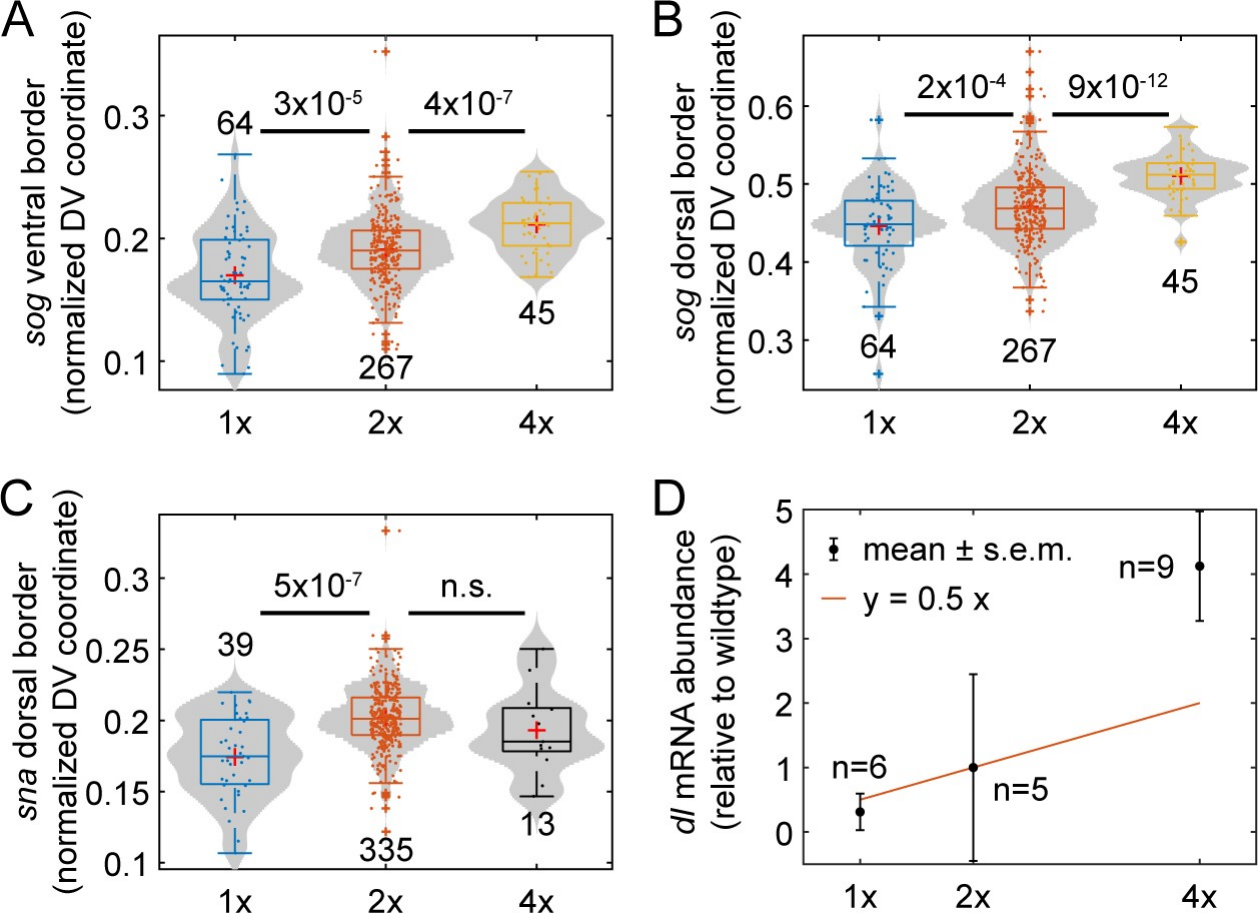
Varying the maternal *dl* dose influences gene expression. (A) Box-and-violin plot of the ventral border of *sog*. (B) Box-and-violin plot of the dorsal border of *sog*. (C) Box-and-violin plot of the of the dorsal border of *sna*. The numbers above or below distributions indicate sample size (numbers of embryos imaged). Numbers between distributions indicate p-value; n.s. = “not significant”. Plus signs indicate statistical outliers. (D) Abundance of *dl* mRNA relative to wildtype, measured by qPCR. Red curve indicates expectation of *y* = 0.5*x*. Circles indicate weighted mean and errorbars indicate weighted standard error of the mean (see Methods). Numbers indicate sample size, including both biological and technical replicates (see Methods).

While the dosage-scaling model predicts high sensitivity of gene expression, limited measurements of gene expression in 1x and 4x embryos [5,26], as well as their viability [27–29], suggest the system is robust. To more accurately quantify the robustness of Dl target gene expression with respect to *dl* dosage, we performed large sample size measurements (generally n ∼ 40 or greater; here and elsewhere in the paper, samples represent biological replicates) of the expression of two Dl target genes, *sna* and *sog*, in 1x, 2x, and 4x embryos. We found that, with only one exception, the expression domains of both genes in 1x and 4x embryos were statistically different from their expression in 2x (wildtype) embryos (p-val ≤ 2× 10^−4^; Fig 3A–3C). The lone exception, the *sna* border in 4x embryos, had a much smaller sample size than the rest (n = 13). Furthermore, the direction of the shifts in gene expression boundaries were as one might expect: in 1x embryos, the gene expression domains shifted closer to the ventral midline, while in 4x embryos, they shifted more dorsally.

Even though we were able to measure statistically significant differences from wildtype, the shifts in gene expression borders were minimal (roughly 10% or less; see Table 1), in contrast to the predictions of the dosage-scaling model (Eq 1). To compare directly with the dosage-scaling model, we calculated the sensitivity coefficient of the three gene expression borders. Mathematically, sensitivity coefficients are calculated by differential changes. Experimentally, one may estimate the sensitivity coefficient by the slope of the log-log plot of the output vs. the input. Using this procedure, we calculated the sensitivity coefficients to be between 0.10 and 0.17 (see Table 1), which are considerably lower than the expectation from the dosage-scaling model.

**Table 1:** Average gene expression locations or Dl gradient widths in 1x, 2x, and 4x embryos. The percent columns are the percent change from wildtype. Sensitivity coefficients are the best-fit slopes of the log-log plot, plus or minus the 68% confidence interval [37].

Property	1x	1x (%)	2x (wt)	4x	4x (%)	Sens. coeff.
*sna* boundary	0.17	14	0.2	0.19	4	0.14 ± 0.03
*sog* ventral boundary	0.17	11	0.19	0.21	10	0.17 ± 0.02
sog dorsal boundary	0.45	5	0.47	0.51	8	0.10 ± 0.01
Dl gradient width	0.13	15	0.15	0.17	11	0.21 ± 0.01

Given the robustness of gene expression, we performed qPCR on *dl* to determine whether the relative abundances of *dl* mRNA loaded into 1x, 2x, and 4x embryos were within expectations (Fig 3D). We found that, relative to wildtype (which had a standard error of 1.4), 1x embryos had an abundance of 0.31 ± 0.28, and 4x embryos had an abundance of 4.12 ± 0.85 (weighted mean ± s.e.m.; see Methods). Thus, it appears the robustness of gene expression domains arises downstream of maternal loading of *dl* mRNA. Together, these measurements suggest that a mechanism exists to mitigate the effects of altering the dosage of maternal *dl* mRNA. We next quantified the Dl gradient itself in 1x, 2x, and 4x embryos.

### Robustness of the Dl gradient

**Fig 4:**
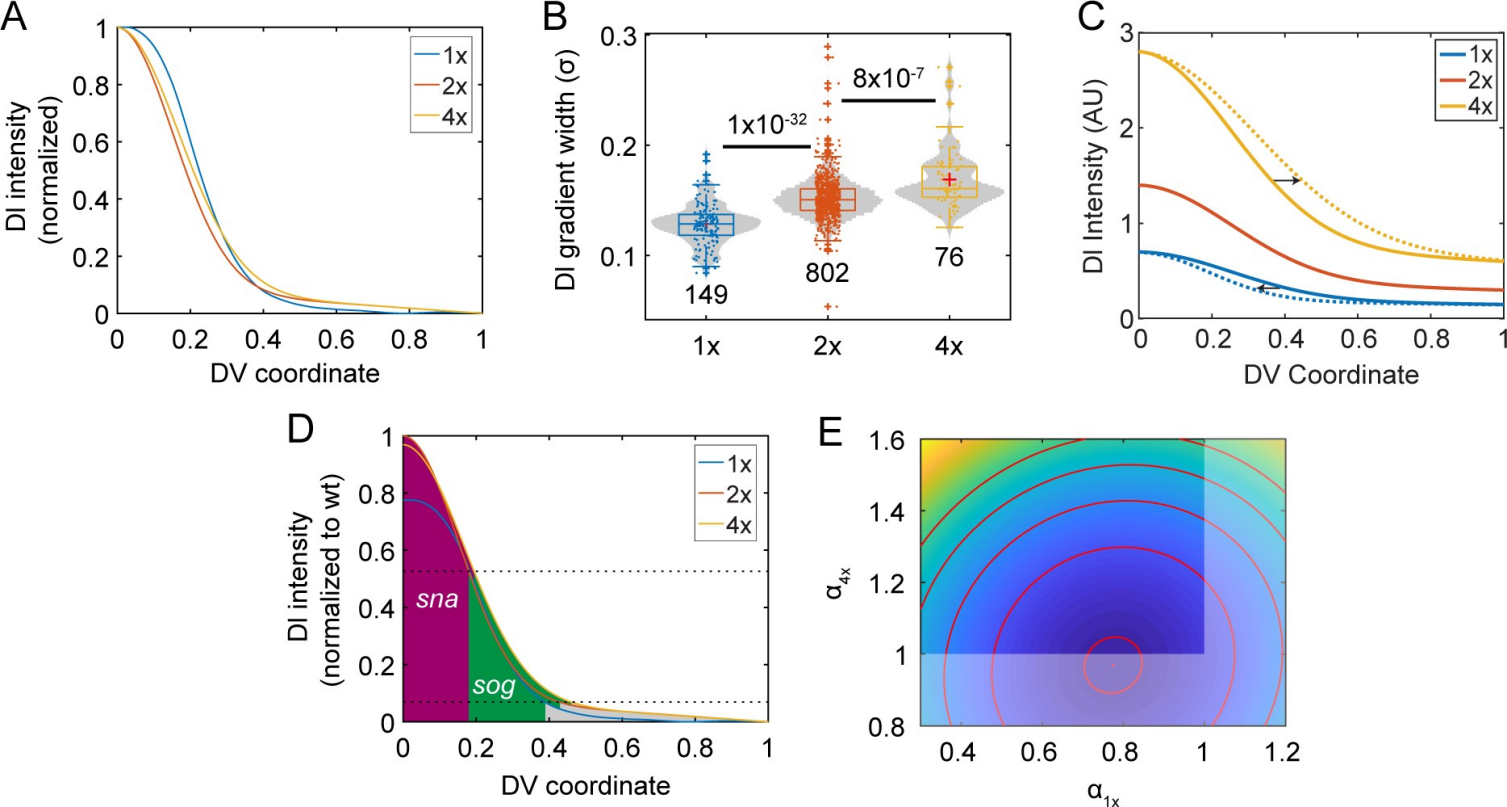
Varying the maternal *dl* dose influences the Dl gradient. (A) Averaged and normalized Dl gradients in 1x, 2x, and 4x embryos. Averaged from n > 30 embryos each (see Methods). (B) Box-and-violin plot of the width of the Dorsal gradient in the genotypes shown in (A). Numbers below distributions indicate sample size. Numbers above indicate p-values. The width of the 1x gradient was modified, as the shape was non-Gaussian (see Supplementary Methods). (C) Dl gradient plot in 1x, 2x, and 4x embryos in the dosage-scaling model showing the effect of higher width in 4x embryos and lower width in 1x embryos. (D) Graph of Dl gradients with best-fit amplitudes for the 1x and 4x gradients, with respect to the 2x gradient set to amplitude of one. (E) Contour plot of the SSE with respect to the amplitude of the 1x gradient (*α*_1*x*_) and that of the 4x gradient *α*_4*x*_. Red dot: the set of best-fit amplitudes. Red curves show the contours of the objective function landscape. Dimmed portion of the *α*_1*x*_,*α*_4*x*_ plane: infeasible region, as realistically, *α*_1*x*_ cannot be greater than 1, and *α*_4*x*_ cannot be less than one.

Previously, it has been shown that a half maternal dose of *dl* significantly shortens and widens the Dl gradient, and results in a flattened, and sometimes double-peaked, top [5,24–26]. Given that this outcome cannot be predicted by the non-robust dosage-scaling description, in which the width and shape of the gradient do not change with dosage, we asked whether such changes to the Dl gradient would be sufficient to confer robustness to predicted gene expression. Therefore, we measured the Dl gradient in embryos loaded with 1x, 2x, and 4x copies of maternal *dl* (see Fig 4A, with average Dl gradients normalized to show differences in shape and width, and Table 1). As previously reported, 1x embryos had a wider and flatter Dl gradient [5,26]. However, the previously-reported width measurements for 1x embryos cannot be directly compared to widths in wildtype embryos, given the width measurements are based on the assumption that the Dl gradient is Gaussian-shaped, which the 1x Dl gradient is not. Accounting for the differing shape (see Supplementary information), the 1x Dl gradient measures as narrower than wildtype (Fig 4B).

When we examined 4x embryos, we found the gradient was statistically wider than wildtype embryos (Table 1; Fig 4A and 4B), which also defies a dosage-scaling description of the Dl gradient. The widening did not appear to be due to peculiarities of the transgenic copies of *dl*, as an alternate formulation of 2x embryos–those with one copy of endogenous *dl* and one transgenic copy (see Methods)–had the same Dl gradient width as wildtype (S1 Fig in S1 File). Even so, the changes to the Dl gradient width are marginal, and the sensitivity coefficient with respect to changes in the dosage is 0.21 ± 0.01 (Table 1). However, rather than explaining the robustness of Dl-dependent gene expression, these measurements naïvely predict even higher sensitivities than the dosage-scaling model. Consider the basic expectation that the 1x gradient should have a roughly 50% lower amplitude than wildtype, while the 4x gradient should have a roughly 200% higher amplitude, even if the gradients are not the exact shape and width as wildtype. The combination of decrease in gradient amplitude and decrease in gradient width in 1x embryos, or an increase of both in 4x embryos, would likely result in sensitive Dl-dependent gene expression, as the two effects (amplitude and width) exacerbate each other (Fig 4C). In contrast, the dosage-scaling model has only one effect: a changing gradient amplitude.

One way to explain the robustness of gene expression, given the observed changes in Dl gradient shape and width, would be if the amplitudes of the 1x and 4x gradients significantly departed from expectation. Therefore, using the average Dl gradients depicted in Fig 4A, we computed the optimum amplitudes for the 1x and 4x gradients (*α*_1*x*_ and *α*_4*x*_, respectively; the 2x amplitude was set to one) that would most closely predict, in the least squares sense, the experimentally observed gene expression of *sna* and *sog* (Fig 4D; see Supplementary Methods). We found that *α*_1*x*_ = 0.78 and *α*_4*x*_ = 0.96 minimizes the sum of the squared errors (SSE) between the predicted and experimentally-observed robust gene expression. However, it is unlikely the 4x gradient would have a lower amplitude than the 2x gradient (dimmed areas in Fig 4D). Therefore, we further varied *α*_1*x*_ and *α*_4*x*_ away from optimum and calculated the SSE between the experimentally-measured and predicted gene expression boundaries (see Supplementary Methods). We found that values slightly greater than one for the 4x amplitude are also acceptable (Fig 4E).

These results suggest that the mechanism to impart robustness with respect to morphogen dosage can control the width, shape, and amplitude of the Dl gradient. Our previous work has shown that facilitated diffusion, also known as shuttling, combined with saturation of the active Toll receptor, can produce the wider, flatter gradients observed in the 1x embryos [26]. Given the saturation of the active Toll receptor, this same mechanism may allow for negligibly taller Dl gradients in 4x embryos. Furthermore, we have seen that 1x embryos that also have compromised shuttling are non-viable [26]. Therefore, to test whether such a combination of mechanisms–shuttling and Toll saturation, together with deconvolution (Fig 2C)–can grant the Dl gradient system robustness with respect to maternal *dl* dosage, we analyzed a mechanistic model capable of capturing these mechanisms.

### Computational modeling of Dl gradient sensitivity

**Fig 5:**
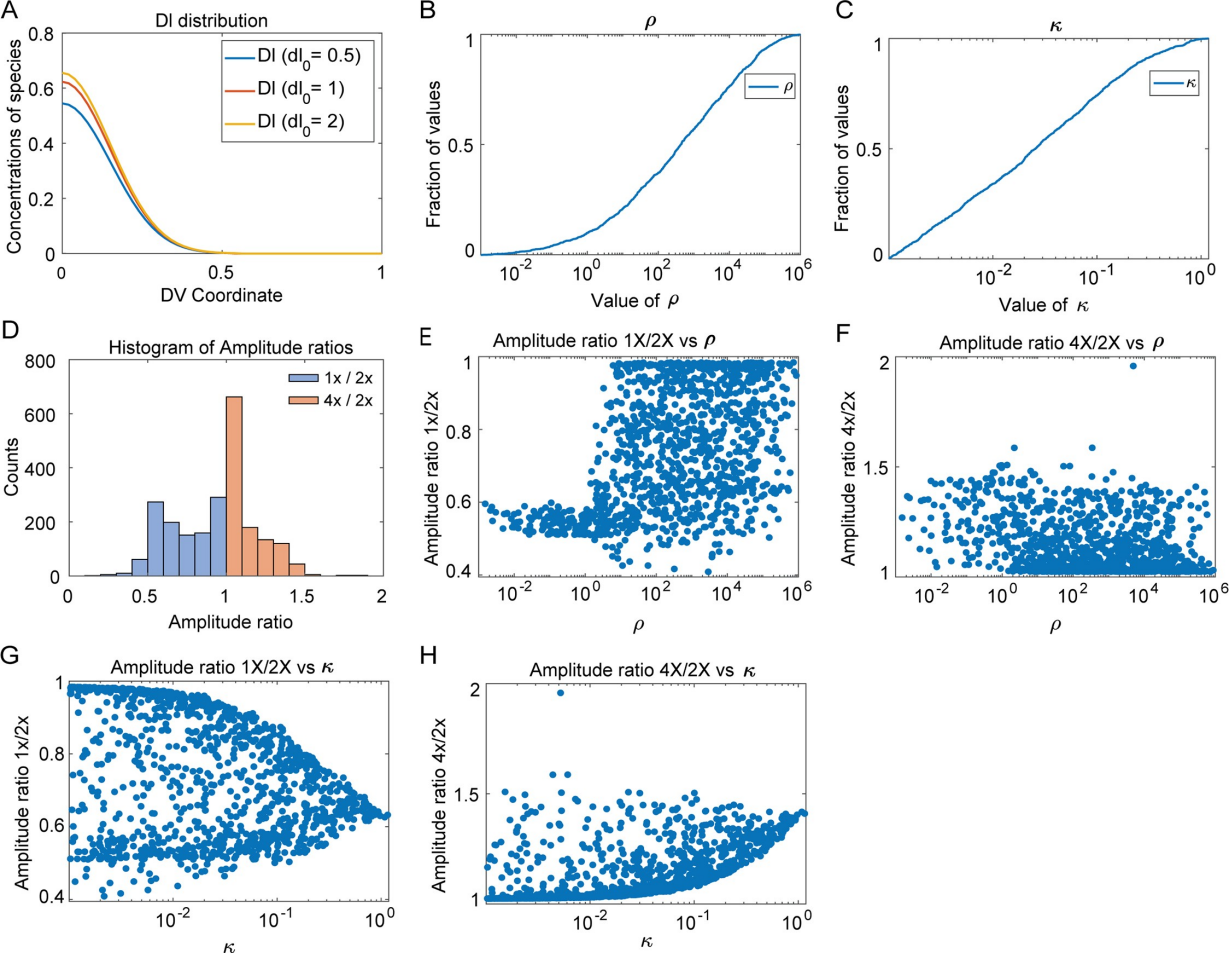
Computational results. (A) Concentration distribution of free Dl for one of the robust parameter sets for dosage 1x,2x and 4x. (B) Cumulative distribution plot for length scale ratio (*ρ*). (C) Cumulative distribution plot of the Michaelis Menten constant (*κ*). (D) Histogram of amplitude ratios. (E) Plot of amplitude ratio 1x/2x against length scale ratio. (F) Plot of amplitude ratio 4x/2x against length scale ratio. (G) Plot of amplitude ratio 1x/2x against *κ*. (H) Plot of amplitude ratio 4x/2x against *κ*.

The model of Dl/Cact interactions analyzed here is based on previous models of the Dl gradient [22,24,26,30]. In particular, we assume that Dl, Cact, and Dl/Cact complex can bind, diffuse, and enter and exit the nucleus; and that Toll signaling can be modeled using a Michaelis-Menten-like formulation (see Methods and Eqs 2 and 3) [26]. Using this model, we performed a random parameter search to screen for parameter sets in which the Dl nuclear gradient was robust to changes in maternal *dl* dosage (Fig 5, see Methods for more details).

We found three trends in the parameter sets necessary for robustness. First, all robust parameter sets predicted that the free Dl nuclear intensity drops to near zero on the dorsal side of the embryo, a result consistent with the deconvolution hypothesis that suggests that Dl fluorescence, as observed in immunostaining experiments or in live embryos expressing Dl-GFP, represents both free Dl and Dl/Cact complex, and that it is important to distinguish between the two [22]. This observation may be similar to the result seen in Fig 2C, in which decay of the Dl gradient to zero at the dorsal midline improved robustness. Fig 5A shows the concentration gradient of free Dl for all three values of dosage (1x, 2x, 4x) for one of the robust parameter sets; it can be seen that all concentration curves fall to zero around lateral regions of the embryo. These same parameter sets predict a gradient of Dl/Cact complex that is non-zero at the dorsal midline (S2 Fig in S1 File), suggesting that, in these simulated embryos, direct fluorescence measurements (the sum of Dl and Dl/Cact in the nucleus) would reveal what appears to be a non-robust Dl gradient. Furthermore, the model does not universally predict that the Dl gradient decays to zero at the dorsal midline. While all robust parameter sets do so, many rejected parameter sets do not (S3 Fig in S1 File). Thus, the model results strongly suggest deconvolution is necessary for robustness.

Second, we found that the effective diffusivity of Dl/Cact complex is greater than that of free Dl in nearly all robust parameter sets. As the flux of Dl/Cact complex is ventrally directed (i.e., shuttling), this result implies that there is a net flux of Dl from dorsal to ventral regions [26]. We plotted the distribution of the ratio of the effective diffusivity of Dl/Cact complex to that of free Dl, henceforth called ratio of length scales (*ρ*), in Fig 5B. In over 95% of the robust parameter sets, this ratio was found to be greater than one. Thus, the constraint of robust gene expression rejects most parameter sets that do not entail facilitated diffusion of Dl by Cact.

Finally, the model also suggests that saturation of Toll receptors is necessary for robustness of gene expression. Since the concentrations are of order 1, a value of the Michaelis Menten constant κ<1 would ensure that Toll receptors are saturated (see Eqs 2 and 3). In all robust parameter sets, the saturation constant for Toll signaling, *κ*, was between 0.001 and 2. Indeed, as seen in Fig 5C, *κ* was the most tightly constrained parameter, which implies tight regulation of Toll saturation may be the most important aspect of the mechanism to ensure robustness of gene expression. This shows that the constraints in the model overwhelmingly favor saturation of Toll receptors by the Dl/Cact complex, as the concentration of Dl/Cact complex in our equations has been scaled to be of order 1. Taken together, these modeling results suggest that the mechanisms of deconvolution and Toll saturation are necessary for a robust DV system, while shuttling of Dl by Cact greatly improves the chances of robustness [22,26].

### Model predictions of amplitude ratios

In addition to showing that the above three mechanisms are required for robustness, the model makes predictions regarding the ratios of amplitudes of the 1x and 4x embryos to that of the wildtype (2x). The ratio of amplitudes of 1x embryos:2x embryos is favored to be greater than 0.5 but less than 1, and that of 4x embryos:2x embryos is favored to fall between 1 and 1.55 (Fig 5D–5H).

The model predicts that the extent of shuttling of Dl by Cact also affects the acceptable values of the amplitude ratios. We observed differences in the distribution of amplitude ratios, for both 1x and 4x embryos, when facilitated diffusion by Cact does not occur (about 5% of parameter sets). For 1x embryos, when *ρ*<1, the values of amplitude ratios are tightly constrained about 0.57, whereas when *ρ*>1 there is larger spread of values (Fig 5E). For 4x embryos, when *ρ*<1, the values of amplitude ratios are slightly constrained and tend to spread towards 1.5 and when *ρ*>1, the values cluster around 1 (Fig 5F). Thus, when shuttling of Dl by Cact does not occur, a smaller range of amplitude ratios are accessible to the embryo which widens when shuttling does occur.

We then investigated the effect of Toll saturation on amplitude ratios. It can be seen from Fig 5G and 5H that as the value of *κ* decreases (and thus, Toll becomes more saturated), the range of amplitude ratios available to robust descriptions of the Dl gradient increases. For lower values of *κ*, a range of 0.5 to 0.85 for ratio of amplitudes of 1x embryos:2x embryos and a range of 1.1 to 1.6 for ratio of amplitudes of 4x embryos:2x embryos is accepted by the model. At higher values of *κ*, the values of the amplitude ratios for 4x/2x and 1x/2x seem to converge to 1.45 and 0.57 respectively, meaning that as Toll is more easily saturated (lower values of *κ*), the model allows for a small, but noticeable range of amplitude ratios for varying dosages. This result seems to indicate that under constrained conditions of Toll saturation, only particular peak amplitudes are preferred–about 1.55 times the wildtype value for 4x embryos and about 0.57 times the wildtype value for 1x embryos. However, if Toll receptors saturate easily, an appreciable range of amplitude ratios leads to robustness. Thus, Toll saturation seems to be an inherent mechanism for robustness in the embryo.

Thus, it seems that both Toll saturation and shuttling of Dl from dorsal to ventral regions allows the embryos to explore a wider range of amplitude ratios, which allows greater flexibility for robustness. However, when the above mechanisms are constrained, the amplitude ratios must take on specific values, which in turn makes it difficult to achieve robustness.

### Changes in Dl gradient amplitude

**Fig 6:**
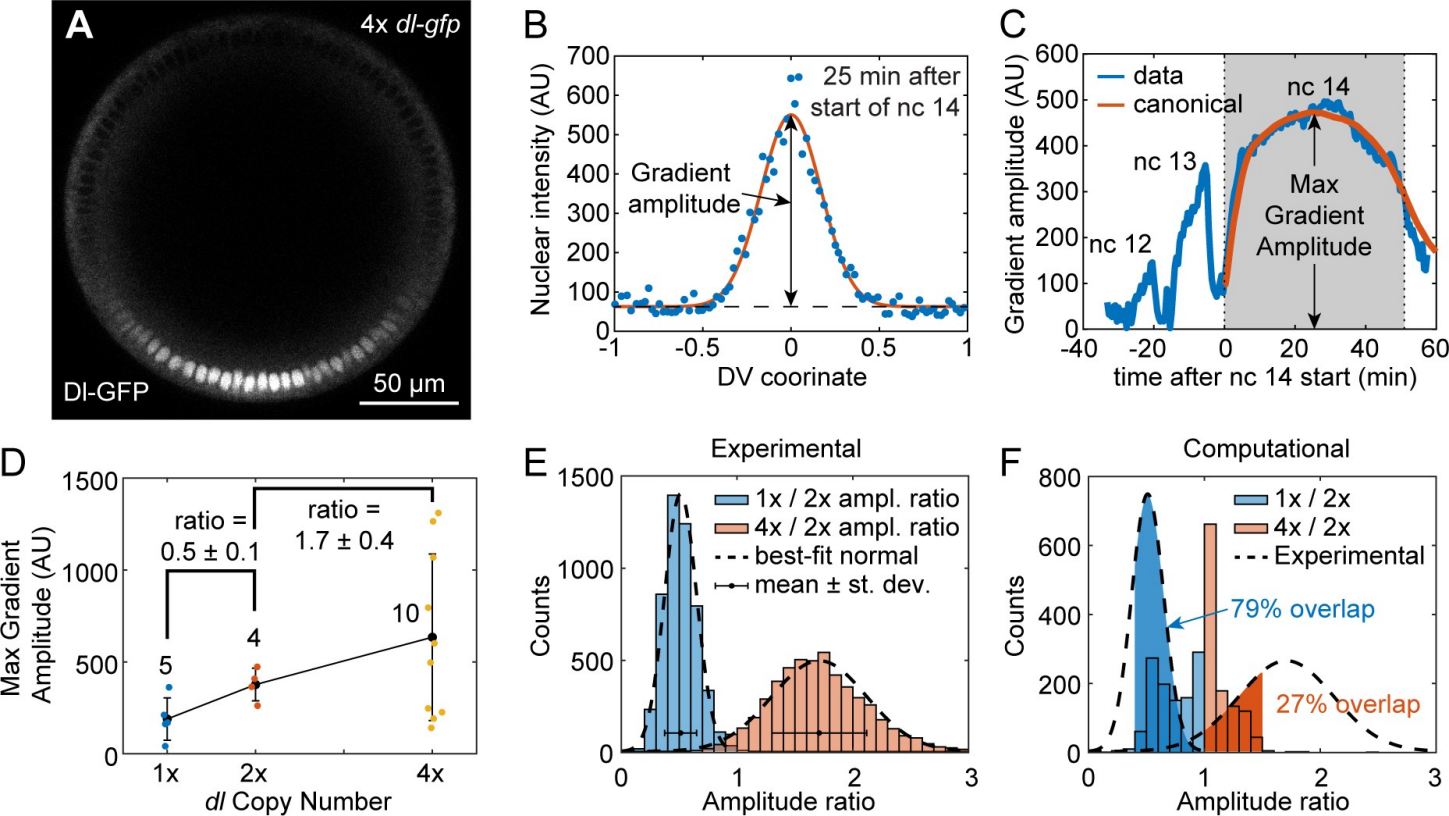
The effect of *dl* dosage on gradient amplitude in live embryos. (A) Cross sectional view of a live *Drosophila* embryo, showing the accumulation of Dl-GFP in the ventral nuclei during late nc 14. (B) Quantification of Dl gradient in live embryos 25 minutes after the start of nc 14. (C) A quantification of the gradient amplitude over time from nc 12 to 14. A canonical curve of gradient amplitude dynamics during nc 14 is plotted in orange. (D) Plot of gradient amplitude (corresponding to the max amplitude during nc 14; see part (C)) of 1x, 2x, and 4x live embryos. The ratios are average plus/minus standard deviation, which were calculated by bootstrap. Whole numbers next to average data points indicate sample sizes. (E) Distributions of amplitude ratios calculated by bootstrap. The normal distributions with the same mean and standard deviation are plotted on top in dashed curves. Mean and standard deviation are depicted on the histogram as dot with errorbars. (F) Comparison of distributions of amplitude ratios obtained computationally (see Fig 5D) and experimentally (dashed black curves; from part (E)). The probability that the experimental 1x:2x amplitude ratio falls between 0.4 and 1 is 0.79 (shaded blue area). The probability that the experimental 4x:2x amplitude ratio falls between 1 and 1.5 is 0.27 (shaded red area).

To test the model predictions of the Dl gradient amplitude needed for robustness, we imaged optical cross sections of live embryos carrying either 1, 2, or 4 copies of Dl-GFP and zero non-tagged versions of Dl (Fig 6A) [34]. The measured Dl-GFP nuclear gradient at each time point was fit to a bell-shaped curve (Fig 6B), which allowed us to measure the gradient amplitude as a function of time. As the Dl gradient exhibits rapid dynamics, live embryo imaging allowed us to consistently measure the largest gradient amplitude (see Methods and Fig 6C). In addition, using live embryos allowed us to overcome several issues that plague fixed, immunostained embryo measurements, including variability in staining intensity and in tissue depth (see Methods).

As expected, the peak Dl gradient amplitude was, on average, lowest for embryos carrying 1 maternal copy of *dl-GFP* (1x *dl-gfp* embryos), intermediate for 2x *dl-gfp* embryos, and highest for 4x *dl-gfp* embryos (Fig 6D). However, the 4x *dl-gfp* embryos exhibited wide variability (Fig 6D). Using bootstrap resampling, we calculated distributions of the amplitude ratios expected from our live imaging data (Fig 6E). We found that 1x *dl-gfp* embryos had roughly one half the gradient amplitude of 2x *dl-gfp* embryos (amplitude ratio = 0.5 ± 0.1), while 4x *dl-gfp* embryos had slightly less than double the gradient amplitude of 2x *dl-gfp* embryos (amplitude ratio = 1.7 ± 0.4). For comparison, our parameter screen predicted robust systems would be likely to have a 1x:2x ratio of greater than 0.5; however, a ratio of 0.5 ± 0.1 was still consistent with many robust parameter sets (Fig 6F). For the 4x:2x amplitude ratio, the parameter screen predicted that a 4x:2x ratio of less than 2 was absolutely required for robustness, which is in line with our experimental results of 1.7±0.4 (Fig 6F).

To quantify the extent of overlap between the experimental measurements and the computational parameter screen, we calculated the probability that the experimentally-measured amplitude ratios would fall within a range that agrees with the distribution of computationally-found amplitude ratios. For the 1x:2x ratio, the range was between 0.4 and 1, and the probability that a random variable, drawn from a normal distribution with the same mean and standard deviation as the experimentally-measured 1x:2x ratio, would fall within this range was 0.79 (Fig 6F). Similarly, for the 4x:2x ratio, the range was between 1 and 1.5, and the probability was 0.27 (Fig 6F). Therefore, while the overlap was not perfect, the experimental measurements were largely consistent with the model’s predictions of robust systems.

## Discussion

Animal development is a complex process that must be buffered against a myriad of environmental, nutritional, and genetic perturbations. The robustness of development with respect to these perturbations often requires regulatory mechanisms. Here we investigated the robustness of gene expression in the early *Drosophila* embryo with respect to variations in the maternal gene dosage of the NF-κB transcription factor Dorsal in a quantitative and computational manner. The NF-κB pathway is highly conserved and is centrally involved in a diverse array of cellular processes, including inflammation, apoptosis, and innate immunity. In flies, Dl/NF-κB also directs embryonic development and differentiation. However, essential questions related to NF-κB robustness in *Drosophila* remain unresolved. Our analysis of an empirical, dosage-scaling description of the Dl gradient, together with detailed measurements of the Dl gradient and its target genes, suggest that a mechanism to control the shape, width, and amplitude of the Dl gradient is necessary for robustness. Our previous work found three novel mechanisms in the establishment of the Dl gradient: deconvolution, shuttling, and Toll saturation [22,26]. In this paper, we used a computational model to study the importance of each of these mechanisms for the robustness of the Dl system.

Recent work showed the importance of deconvolving experimentally-measured fluorescence signal into free Dl and bound Dl (Dl/Cact complex) when interpreting the Dl gradient [22]. Doing so results in a nuclear Dl gradient that drops to near zero instead of to non-zero basal levels at dorsal regions [5,15,20,21]. In the dosage-scaling model, deconvolution was modeled by setting basal levels to near zero. While this choice of basal levels improved robustness somewhat in the dosage-scaling model, the gene expression boundaries remained overly sensitive to *dl* dosage, which indicated that deconvolution by itself is not sufficient for robustness. However, deconvolution appears to be necessary: every robust parameter set in the computational model predicted a free Dl gradient that decayed to near zero, whereas non-robust parameter sets did not.

Our model also suggests the shuttling mechanism increases robustness of the Dl system. In such a mechanism, Toll signaling creates a sink for Dl/Cact complex, which establishes a ventrally-directed flux to accumulate Dl in ventral regions. While it is possible that free Dl then diffuses dorsally, such counter-diffusion is likely mitigated by capture of free Dl by the nuclei. Previous work in our lab suggests that shuttling of Dl/Cact complex from dorsal to ventral regions is an important factor for robustness in the embryo [26]. Our model supports this result, as most parameter sets that selected for robust gene expression favored facilitated diffusion of Dl by Cact, as the effective diffusivity of Dl/Cact was higher than that of free Dl.

Previous work also suggested that, in wildtype embryos, active Toll receptors are limiting [26], thereby maintaining robust gene expression, even when *dl* dosage varies from wildtype. In wildtype embryos, when active Toll signaling complexes are saturated with Dl/Cact complex, a significant number of Dl/Cact complexes bypass the ventral-lateral regions without being dissociated, and Dl is shuttled to the ventral-most portions of the embryo. On the other hand, if active Toll signaling complexes are not saturated, as may be the case in 1x embryos, the Dl/Cact complex will be dissociated at a higher rate in the ventral-lateral regions of the embryo and will be unable to reach the ventral-most regions of the embryo. The lack of Toll saturation in 1x embryos thus results in a flatter and wider concentration gradient of nuclear Dl.

One interesting aspect of the model is that the Dl gradient amplitude does not perfectly scale with dosage. The model predicts that, in robust systems, the ratio of amplitudes in 1x vs 2x embryos is between 0.5 and 1, while the ratio of amplitudes in 4x vs 2x is between 1 and 1.5. This phenomenon may also be related to Toll saturation. While embryos with 4 copies of dl have double the wildtype Dl dose, twice as much Dl will not necessarily enter the nuclei because that process relies on Toll signaling, which may be saturated. Similarly, decreasing the *dl* dosage, as in the case of 1x embryos, implies halving the amount of Dl/Cact complex without halving the absolute number of free Dl molecules that will enter the nuclei. Thus, if the active Toll complexes remain constant in all three cases of dosage and provided that they are saturated, the 1x:2x Dl gradient amplitude ratio may be greater than 0.5, and the 4x:2x ratio may be significantly less than 2 (Fig 5A). Our live imaging results, in which we measured the Dl gradient amplitudes in live embryos expressing varying dosages of *dl-gfp*, were largely consistent with the model predictions.

In this work we have demonstrated the importance of certain built-in mechanisms within the early *Drosophila* embryo that ensure robustness of gene expression along the DV axis. These three mechanisms, (deconvolution of the measured Dl fluorescence into free Dl and Dl/Cact complex, saturation of Toll receptors by Dl/Cact complex, and shuttling of Dl by Cact from dorsal to ventral regions of the embryo) are crucial for ensuring that genes expressed in the DV axis have domain boundaries in specific regions. We have presented both experimental and computational evidence that these processes are paramount for safeguarding against genetic perturbations to *dl* dosage. The advances in studying the molecular mechanism behind robustness with respect to maternal *dl* dosage may open the door for understanding the question of how sustained embryonic development can be achieved despite genetic and environmental fluctuations.

## Methods

### Fly stocks

For fixed embryo imaging and qPCR, the laboratory stock *yw* was used as wildtype (2x), 1x embryos were dl^1^ cn^1^ sca^1^/CyO, l(2)DTS100^1^ (Bloomington #3236), and 4x embryos were dl^RC^/dl^RC^; [26]. The alternate 2x embryos (S1 Fig in S1 File) were generated by crossing dl^4^ pr^1^ cn^1^ wx^wxt^ bw^1^/CyO (Bloomington #7096) with the dl^RC^/dl^RC^ line to arrive at dl^4^ pr^1^ cn^1^ wx^wxt^ bw^1^/+; dl^RC^/+.

For live imaging, 1x embryos were dl^1^, dl-mgfp, H2A-rfp/dl^1^. The dl-mgfp construct was previously reported [26]. The 2x embryos were dl^1^, dl-mgfp, H2A-rfp/dl^1^, dl-mgfp. The 4x embryos were dl^1^, dl-mgfp, H2A-rfp/dl^1^, dl-mgfp; dl-mgfp /dl-mgfp, which included an insertion of the dl-mgfp construct onto the third chromosome. The H2A-rfp was recombined from w[*]; P{w[+mC] = His2Av-mRFP1}II.2 (Bloomington #23651).

### Fluorescent in situ hybridization and fluorescent immunostaining

Standard protocols to detect Dl and Histone protein and *sna* and *sog* mRNA were followed. All embryos were aged to NC 14 (approx. 2–4 hours after egg lay), then fixed in 37% formaldehyde according to standard protocols [35]. A combination fluorescent *in situ* hybridization/fluorescent immnuostaining was performed according to standard protocols [35]. Briefly, fixed embryos were washed in PBS/Tween and hybridized at 55 ^o^C overnight with anti-sense RNA probes, which were generated according to standard lab protocol. The embryos were then washed and incubated with primary antibodies at 4 ^o^C overnight. The next day, they were washed and incubated for 1–2 hrs with fluorescent secondary antibodies at room temperature. The embryos were then washed and stored in 70% glycerol at -20 ^o^C. Embryos were imaged within one month of completing the protocol.

Antibodies used were anti-dorsal 7A4 (deposited to the DSHB by Ruth Steward (DSHB Hybridoma Product anti-dorsal 7A4)) (1:10), donkey anti-mouse- 488 (Invitrogen A21202, Lot 81493) (1:500), rabbit anti-histone (abcam ab1791, Lot 940487) (1:5000), donkey anti-rabbit-546 (Invitrogen A10040, Lot 107388) (1:500), goat anti-biotin (ImmunoReagents, Raleigh, NC, GtxOt-070-D, Lot 19-19-112311) (1:50,000), donkey anti-goat-647 (Invitrogen A21447, Lot 774898) ((1:500), goat anti-fluorescin (Rockland 600-101-096, Lot 19458) (1:500), rabbit anti-fluorescin (Life Technologies A889, Lot 1458646) (1:500), goat anti-histone (Abcam, ab12079, Lots GR6952-4 and GR129411-1) (1:100), donkey anti-rabbit-350 (ImmunoReagents, DkxRb-003-D350NHSX) (1:500). For some experiments the nuclear stain Draq5 (Cell Signaling #4084S) was used instead of an anti-histone antibody.

### Mounting and imaging of fixed embryos

Embryos were cross sectioned and mounted in 70% glycerol as described previously [34]. Briefly, a razor blade was used to remove the anterior and posterior thirds of the embryo, leaving a cross section roughly 200 μm long by 200 μm in diameter. Previous work has shown the Dl gradient is roughly constant within such a cross section [20]. These sections were then oriented such that the cut sides became the top and bottom. Sections were then imaged at 20x on a Zeiss LSM 710 microscope. 15 z-slices 1.5 μm apart were analyzed, for a total section size of 21 μm.

### Image analysis of fixed embryos

Images of embryo cross sections were analyzed using a previously derived algorithm [36]. Briefly, the border of the embryo was found computationally, then the nuclei were segmented using a local thresholding protocol. The intensity of Dl in each segmented nucleus was calculated as the ratio between the intensity in the Dl channel divided by the intensity in the nuclear channel. The intensity of mRNA expression was calculated as average intensity within an annulus roughly 18 μm wide around the perimeter of the embryo.

mRNA profiles were fit to canonical profiles, which have been previously derived from averaging at least 10 gene expression profile measurements as described in [36]. The fitting procedure results in identifying the amplitude, background levels, and width of each *sna* and *sog* peak. Additionally, for *sog*, the location of the peak is determined as well. The border(s) of the two genes are then taken as the half-max locations of the canonical profiles with the same location and width as the fitted peaks. See ref [36] for more details. Gene expression profiles with a goodness of fit (gof) less than 0.8 were omitted from study. Our results are robust to choice of gof cutoff, as changing this threshold to 0.7 or 0.9 does not alter our conclusions.

All Dl gradients were fit to a Gaussian, and these fits were used to determine the width parameter, σ. Gradients with a gof less than 0.7 were eliminated from the results. Our results are robust to choice of gof cutoff, as changing this threshold to 0.8 or 0.9 does not alter our conclusions. Normalized intensity plots were generated by fitting each embryo’s data to its own Gaussian by subtracting the B value and 70% of the M value, then dividing by the A value. (X = (x–B– 0.7M)/A)).

The average normalized intensity plot of the Dl gradients (Fig 4A) was generated by averaging the normalized intensity plots of a large number of embryos in the specified genotype. In the averaging process, the curves were first aligned according to the predicted ventral midline, then they were normalized according to the procedure above, then the arithmetic mean was taken at each point along the DV axis, from x = -1 to 1 with 301 points. For 1x *dl* embryos, n = 63. For 2x *dl* embryos, n = 33. For 4x *dl* embryos, n = 34.

The data in Figs 3 and 4 were pooled from multiple experiments performed on multiple dates. Each experiment using 1x *dl* or 4x *dl* embryos was performed alongside wildtype controls. The ensemble of Dl gradient widths or *sna*/*sog* gene expression domains for each wildtype control were not statistically distinguishable at an alpha-level of 0.05 from experiment to experiment. Statistical significance was calculated using two-tailed homoscedastic t-tests.

### Mounting and imaging of live embryos

Mounting of embryos is described in [34]. Briefly, 2 hour old embryos (nc 14) were collected from cages, dechorionated with bleach for 30 seconds, and mounted on a 22 mm square cover slip that was broken in half and treated with heptane glue. The cover slip was then attached with a piece of double-sided tape to a mounting block as described in [34]. The anterior-posterior axis of each embryo was oriented perpendicularly to the bottom of the cover slip and placed so that approximately half of the embryo is on the cover slip and half is hanging off. The cover slip was precisely aligned with the top of the 21.5 mm-tall mounting block. This mounting block with cover slip attached was then placed in a glass-bottom petri dish (Matek) containing small amount of DI water.

Before placing the embryos on the microscope, the intensity output of the 488 laser was measured using the transmitted light channel, as described previously [5]. Without a sample, the 488 laser would be unimpeded to pass to the transmitted light channel. This intensity measurement allowed us to control for day-to-day variation in laser intensity. See Supplementary Methods for more detail.

A Zeiss 880 confocal microscope was used to acquire timecourse movies of single z-slices of embryos. Images were taken at a depth of 150 μm from the pole closest to the objective. The laser power was kept lower than 10% to avoid photobleaching and phototoxicity. Images were 512x512 using a scan speed of 7.

### Image analysis of live embryos

Time course images of live embryos were analyzed as follows. First, each image of the Dl-GFP gradient was treated as described above for fixed embryos. Once the gradient amplitude was found for each image in the timecourse, the curve of gradient amplitude vs time, *A*(*t*), was visually inspected for the beginning of nc 14. The embryo image was visually inspected for the start of gastrulation. These two time points demarcate the duration of nc 14 interphase, and for each qualifying embryo timecourse (i.e., the ones that captured both time points), the duration of nc 14 was computed as *T*_*nc*14_.

Next, the *A*(*t*) curves for all qualifying embryos were normalized plotted on top of each other after two normalization procedures. The first procedure was to divide by the max intensity. The second procedure was to stretch the time variable so that each qualifying embryo had an nc14 duration of one hour. This was done by dividing the time variable by the *T*_*nc*14_ corresponding to each individual embryo and multiplying by one hour. Once all *A*(*t*) curves were plotted on top of one another, they were averaged together to obtain a “canonical” nc 14 gradient amplitude curve (see S4 Fig in S1 File). Once the canonical nc 14 curve was obtained, each nc 14 *A*(*t*) curve was fit to the canonical curve to obtain a peak value (in time) of the gradient amplitude. These peak values served as the data plotted in Fig 6D.

### qPCR

qPCR was performed on 1x embryos (dl^1^ cn^1^ sca^1^/CyO, l(2)DTS100^1^; Bloomington #3236), 2x embryos (*yw* laboratory strain), and 4x embryos (dl^RC^/dl^RC^; [26]). RNA collection of embryo samples was performed using a Trizol/chloroform extraction. For each sample, cDNA was created by first treating the RNA samples with DNase I (ThermoFisher Scientific), then using Super Script II Reverse Transcriptase (ThermoFisher Scientific) to make cDNA.

The qPCR primers (Integrated DNA Technologies) for *dl* were designed as follows: forward primer: 5’- TGG CTT TTC GCA TCG TTT CCA G -3’ and reverse primer: 5’- TGT GAT GTC CAG GGT ATG ATA GCG -3’Actin was used as a housekeeping gene to normalize samples. The *actin* primers were designed as follows: forward primer: 5’—CCG TGA GAA GAT GAC CCA GAT C-3’ and reverse primer: 5’- TCC AGA ACG ATA CCG GTG GTA C -3’. The qPCR protocol included an initial denaturation and enzyme activation step for 2 minutes at 95°C, followed by 50 cycles of denaturing at 95°C for 10 seconds, annealing at 60°C for 30 seconds, and extension at 72°C for 30 seconds.

Each qPCR run consisted of all three genotypes, and each genotype had three biological replicates, each of which had three technical replicates to control for pipetting. Thus, in a single qPCR run, there were a total of 54 samples (nine of *dl* per genotype, and nine of *actin* per genotype). Three separate qPCR runs were performed. Each run had the same three biological replicates per genotype.

Δ*C*_*T*_ values were calculated by [software] of the *C*_*T*_ for *dl* minus the *C*_*T*_ for the corresponding *actin* well. Some *dl* samples failed to give a *C*_*T*_ value (see Supplementary S2 File).

Analysis was performed in the following manner. For each genotype *g* and a given qPCR run/biological replicate *i*, the Δ*C*_*T*_ values from the three technical replicates were averaged to give a value *y*_*i*,*g*_, and the standard deviation of the Δ*C*_*T*_ values was calculated to give *s*_*i*,*g*_, where *i* = 1…*n*. (If no Δ*C*_*T*_ values were discarded, *n* would equal 9: three biological replicates repeated over three days. However, instances where there were fewer than two technical replicates that gave a valid Δ*C*_*T*_ value were discarded. The values of *n* for each genotype can be found in Fig 3D). From the means, *y*_*i*,*g*_, and standard deviations, *s*_*i*,*g*_, of the technical replicates, a weighted mean and weighted S.E.M. was calculated for each genotype, *g*, according to:

$$weighted\;mean:\;{\bar{y}}_{g}=\sum_{i=1}^{n}w_{i,g}y_{i,g}$$

$$weighted\;s.e.m.:S_{g}=\sqrt{\frac{1}{n-1}\sum_{i=1}^{n}w_{i,g}{\left(y_{i,g}-\bar{y}\right)}^{2}}/\left(\frac{1}{n\sqrt{n}}\sum_{i=1}^{n}w_{i,g}\right)$$

where the weights *w*_*i*_ are defined by

$$w_{i,g}=\frac{1}{s_{i,g}}/\sum_{j=1}^{n}\frac{1}{s_{j,g}}$$

The values of ${\bar{y}}_{g}$ are plotted in Fig 3D with errorbars of *S*_*g*_ for each genotype *g*.

### Model equations

The equations for the computational model are as follows:

$$\frac{du_{h}}{dT}=a_{1}\lambda_{d}(u_{h-1}-2u_{h}+u_{h+1})+a_{2}\beta (x)\frac{w_{h}}{\kappa +w_{h}}-a_{3}\gamma u_{h}$$ (2)

$$\frac{dw_{h}}{dT}=a_{4}\lambda_{dc}(w_{h-1}-2w_{h}+w_{h+1})-a_{5}\beta (x)\frac{w_{h}}{\kappa +w_{h}}+a_{6}\gamma u_{h}$$ (3)

Where β(x)=βoexp(−xϕ)2 represents the gaussian Toll-mediated rate constant and *κ* represents the Michaelis Menten constant for the dissociation of Dl/Cact complex; *u* and *w* represent cytoplasmic species Dl and Dl/Cact complex respectively; subscript *h* represents a nucleus and its associated cytoplasmic compartment; *λ*_*i*_ represents effective intercompartmental exchange rates; and the *a*_*i*_’s are constant weighting factors related to the nuclear import/export equilibrium constants and the geometry of the nucleus and cytoplasm (see Supplementary Information for more details). The free parameters of the model—*λ*_*u*_,*λ*_*w*_,*β*,*γ* and *κ* –are each varied randomly between 1e-3 to 1e+3. A total of ~200000 parameter sets were obtained of which 1150 satisfied the error criterion, which represented by every point in Fig 5D–5G.

Eqs (2 and 3) above have been derived after simplifying a more detailed model (see Supplementary information for details). The nuclei are modeled as spheres sitting in cuboidal cytoplasmic compartments that span the periphery of the embryo. Since the embryo is approximately symmetric about the DV axis; the spatial coordinate was varied from 0 to 1 with the former representing the ventral midline and the latter, the dorsal midline. The number of such compartments/nuclei/cells is taken to be 51, approximately equal to the number of nuclei in NC 14 found from live fluorescence imaging [20]. Both nuclei and the cytoplasm volumes are considered well mixed. We assume that the nucleus and cytoplasm are in a state of pseudo-equilibrium. Thus, *k*_*out*_*C*_*nuc*_≈*k*_*in*_*C*_*cyt*_ or *C*_*nuc*_≈*K*_*eq*_*C*_*cyt*_ where, *K*_*eq*_≡*k*_*in*_/*k*_*out*_ is defined as the equilibrium constant for nuclear import/export for all species. The effect of Toll was modeled with a Michaelis Menten formulation, assuming the concentration of the intermediate species Dl-Cact-Toll to be approximately constant in nuclear cycle 14. The above equations were then non-dimensionalized, approximately with respect to the conditions found in wildtype *Drosophila* embryos at the beginning of NC 14, such that every term was of order 1. The ratio of effective diffusivities or the length scale ratio was then defined as

$$\rho =\frac{\lambda_{dc}}{{\tilde{V}}_{nuc}K_{eq,dc}+{\tilde{V}}_{cyt}}/\frac{\lambda_{d}}{{\tilde{V}}_{nuc}K_{eq,d}+{\tilde{V}}_{cyt}}$$ (4)

where ${\tilde{V}}_{nuc/cyt}=\frac{V_{nuc/cyt}}{\hat{V_{14}}}$ (see Supplementary information for details).

The simulation was run for 60 min, which approximates the time period of NC 14, which is the longest nuclear cycle of the blastoderm. Dosage was varied by doubling or halving the initial concentration of Dl/Cact. The dimensionless constants obtained from it were then varied from 1e-3 to 1e+3 to obtain concentration profiles for Dl and Dl/Cact. From these concentration profiles, the dorsal border of *sna* and the ventral and dorsal borders of *sog* were calculated assuming the borders are defined by thresholds of free Dl concentration. These model predictions of the borders were compared with experimental values in the least square error sense and parameter sets with errors lower than a set value were accepted as robust (see Supplementary information for details).

### Least squares method for determining robustness in the computational model

The error in the predictions of boundaries of gene expression was defined, for every border, as follows,

$$e_{\beta }(\theta )={\left(\varepsilon_{\beta ,1x}\right)}^{2}+{\left(\varepsilon_{\beta ,2x}\right)}^{2}+{\left(\varepsilon_{\beta ,4x}\right)}^{2}$$

$$={\left(\frac{x_{\beta ,model,1x}(\theta )-x_{\beta ,exp,1x}}{\sigma_{\beta ,exp,1x}}\right)}^{2}+{\left(\frac{x_{\beta ,model,2x}(\theta )-x_{\beta ,exp,2x}}{\sigma_{\beta ,exp,2x}}\right)}^{2}$$

$$+{\left(\frac{x_{\beta ,model,4x}(\theta )-x_{\beta ,exp,4x}}{\sigma_{\beta ,exp,4x}}\right)}^{2}$$ (5)

where, *x*_*β*,*model*,*g*_ is the model boundary prediction, *x*_*β*,*exp*,*g*_ is the experimental measure of border and *σ*_*β*,*exp*,*g*_ is the experimentally observed variation in boundary of gene *β* of genotype g.

For any gene expression border *β*∈*B*, where *B* = {sna, sogd, sogv} and genotype *g*∈*G* where *G* = {1x, 2x, 4x}, the error is calculated by minimizing *e*_*β*_(*θ*) with respect to its concentration threshold *θ*. Those parameter sets with error values less than 1.5 for all gene expression boundaries, were deemed robust.

## Supporting information

S1 File This file contains details of methodology. (DOCX)

S2 File This file contains qPCR results. (XLSX)
