# Supplementary material for: Robustness of the Dorsal morphogen gradient with respect to morphogen dosage
Source: PLoS Comput Biol. 2020 Apr 6;16(4):e1007750. doi: 10.1371/journal.pcbi.1007750 (PMC7162545; doi:10.1371/journal.pcbi.1007750)
Supplement: S1 File — (DOCX) [file pcbi.1007750.s001.docx]

# Detailed computational model

Previous models of the Dl nuclear gradient can be classified in terms of their complexity and the number of realistic features they support. In 2009, Kanodia et al. published pioneering modeling work on the Dl gradient, which captured the establishment of Dl gradient by an interaction between Dl, Cact and Toll in the cytoplasm. It was found that this model was inconsistent with the data from live measurements of Venus-tagged Dl. The only way to work around this inconsistency was to assume the presence of nuclear Cact, thus nuclear Dl/Cact. The following model equations represents the full model for the Dl system, which consists of three species namely, Dl, Cact and Dl/Cact complex that are allowed to move between cytoplasmic compartments and between nucleus and cytoplasm within a cell.

| $\frac{d\left[ V_{nuc}C_{d,nuc}^{h} \right]}{dt}= A_{nuc}\left( k_{in,d}C_{d,cyt}^{h}-k_{out,d}C_{d,nuc}^{h} \right) -V_{nuc}\left( k_{b}C_{d,nuc}^{h}C_{c,nuc}^{h} \right)$ | (1) |
| --- | --- |
| $\frac{d\left[ V_{cyt}C_{d,cyt}^{h} \right]}{dt}= A_{cyt}\Gamma_{d}\left( C_{d,cyt}^{h-1}-2C_{d,cyt}^{h}+ C_{d,cyt}^{h+1} \right) +V_{cyt}\left( \frac{k_{d}\left( x \right)C_{dc,cyt}^{h}}{\kappa+C_{dc,cyt}^{h}}- k_{b}C_{d,cyt}^{h}C_{c,cyt}^{h} \right) -A_{nuc}\left( k_{in,d}C_{d,cyt}^{h}-k_{out,d}C_{d,nuc}^{h} \right)$ | (2) |
| $\frac{d\left[ V_{nuc}C_{dc,nuc}^{h} \right]}{dt}= A_{nuc}\left( k_{in,dc}C_{dc,cyt}^{h}-k_{out,dc}C_{dc,nuc}^{h} \right) + V_{nuc}\left( k_{b}C_{d,nuc}^{h}C_{c,nuc}^{h} \right)$ | (3) |
| $\frac{d\left[ V_{cyt}C_{dc,cyt}^{h} \right]}{dt}= A_{cyt}\Gamma_{dc}\left( C_{dc,cyt}^{h-1}-2C_{dc,cyt}^{h}+ C_{dc,cyt}^{h+1} \right) -V_{cyt}\left( \frac{k_{d}\left( x \right)C_{dc,cyt}^{h}}{\kappa+C_{dc,cyt}^{h}}- k_{b}C_{d,cyt}^{h}C_{c,cyt}^{h} \right) -A_{nuc}\left( k_{in,dc}C_{dc,cyt}^{h}-k_{out,dc}C_{dc,nuc}^{h} \right)$ | (4) |
| $\frac{d\left[ V_{nuc}C_{c,nuc}^{h} \right]}{dt}= A_{nuc}\left( k_{in,c}C_{c,cyt}^{h}-k_{out,c}C_{c,nuc}^{h} \right) -V_{nuc}\left( k_{b}C_{d,nuc}^{h}C_{c,nuc}^{h} \right)$ | (5) |
| $\frac{d\left[ V_{cyt}C_{c,cyt}^{h} \right]}{dt}= A_{cyt}\Gamma_{c}\left( C_{c,cyt}^{h-1}-2C_{c,cyt}^{h}+ C_{c,cyt}^{h+1} \right) +V_{cyt}\left( \frac{k_{d}\left( x \right)C_{dc,cyt}^{h}}{\kappa+C_{dc,cyt}^{h}}- k_{b}C_{d,cyt}^{h}C_{c,cyt}^{h}- k_{deg}C_{c,cyt}^{h} \right) -A_{nuc}\left( k_{in,c}C_{c,cyt}^{h}-k_{out,c}C_{c,nuc}^{h} \right)+P_{c}$ | (6) |

Here, subscripts *nuc* and *cyt* represent nucleus and cytoplasm respectively; *d, c,* and *dc* represent species Dl, Cact, and Dl/Cact complex respectively; superscript *h* represents a nucleus and its associated cytoplasmic compartment. The parameters, $k_{in,species}$ and $k_{out,species}$ represents nuclear import and export rates, $k_{b}$ represents Dl/Cact binding constant, $\Gamma_{species}$ represents intercompartmental exchange rates, $k_{d}\left( x \right)=k_{d}^{max}exp\left( \frac{x}{\phi} \right)^{2}$represents the gaussian Toll-mediated rate constant and $\kappa$ represents the Michaelis Menten constant for the dissociation of Dl/Cact complex, $k_{deg}$ represents the degradation rate constant for Cact and $P_{c}$ represents rate of production of Cact.

The Dl system is represented by 6 equations consisting of Dl, Cact and Dl/Cact in the nucleus and in the cytoplasm. This model is based on previous models used in the literature with some modifications. Firstly, Cact and the Dl/Cact complex were allowed to enter the nucleus and secondly Michaelis Menten kinetics was used to describe the dissociation of the Dl/Cact complex by Toll in the cytoplasm. The width of Toll gradient was fixed at $\phi= 0.15$, which approximates the width of wildtype Dl gradients. In order to minimally describe the effect of dosage of the Dl morphogen on the embryo’s development these equations were simplified based on the following assumptions. Firstly, since the time scales of transport of species between adjacent cytoplasmic compartments is much higher than that of nuclear exchange, a state of pseudo equilibrium is assumed between the nucleus and cytoplasm. Thus, $k_{out}C_{nuc}\approx k_{in}C_{cyt}$ or $C_{nuc}\approx K_{eq}C_{cyt}$ where, $K_{eq} \equiv{k_{in}}/{k_{out}}$ is defined as the equilibrium constant for nuclear import/export. The values for the equilibrium constants are fixed at $K_{eq,d} = 4$, $K_{eq,dc} = 1$ and $K_{eq,c} = 1$ [1]. Secondly, since Cact has a high turnover rate, a uniform concentration of Cact, equal to that at the beginning of nuclear cycle 14 in wildtype embryos, was assumed. Shown below are equations where concentrations have been non-dimensionalized using conditions at the beginning of nuclear cycle 14 in wildtype embryos.

| $\frac{d\left[ \left( V_{nuc}K_{eq,d}+V_{cyt} \right)u^{h} \right]}{dT}=\Gamma_{d}A_{cyt}\left( u^{h-1}-2u^{h}+ u^{h+1} \right) +V_{cyt}\left( \frac{\beta\left( x \right)w^{h}}{\kappa+C_{d}^{wt}w^{h}}-k_{b}C_{d}^{wt}C_{c}^{wt}u^{h}v^{h} \right)-V_{nuc}\left( k_{b}K_{eq,d}K_{eq,c}C_{d}^{wt}C_{c}^{wt}u^{h}v^{h} \right)$ | (7) |
| --- | --- |
| $\frac{d\left[ \left( V_{nuc}K_{eq,dc}+V_{cyt} \right)w^{h} \right]}{dT}=\Gamma_{dc}A_{cyt}\left( w^{h-1}-2w^{h}+ w^{h+1} \right) -V_{cyt}\left( \frac{\beta\left( x \right)w^{h}}{\kappa+C_{d}^{wt}w^{h}}-k_{b}C_{c}^{wt}u^{h}v^{h} \right)+V_{nuc}\left( k_{b}K_{eq,d}K_{eq,c}C_{c}^{wt}u^{h}v^{h} \right)$ | (8) |
| $\frac{d\left[ \left( V_{nuc}K_{eq,c}+V_{cyt} \right)v^{h} \right]}{dT}=\Gamma_{c}A_{cyt}\left( v^{h-1}-2v^{h}+ v^{h+1} \right)-\frac{V_{cyt}}{C_{c}^{wt}}\left( \frac{\beta\left( x \right)C_{d}^{wt}w^{h}}{\kappa+C_{d}^{wt}w^{h}}-k_{b}C_{d}^{wt}C_{c}^{wt}u^{h}v^{h}-k_{deg}C_{c}^{wt}v^{h} \right)+\frac{V_{cyt}}{C_{c}^{wt}}\left( k_{b}K_{eq,d}K_{eq,c}C_{d}^{wt}C_{c}^{wt}u^{h}v^{h} \right)+\frac{P_{c}}{C_{c}^{wt}}$ | (9) |

where,

$u^{h}=\frac{C_{d,cyt}^{h}}{C_{d}^{wt}}$ $w^{h}=\frac{C_{dc,cyt}^{wt}}{C_{d}^{wt}}$ $v^{h}=\frac{C_{c,cyt}^{h}}{C_{c}^{wt}}$ $C_{c}^{wt}=\frac{P_{c}}{k_{deg}}$

Due to the high turnover rate of Cact, equation 9, upon non-dimensionalizing simplifies to $v^{h} = 1$. Finally, the equations in the main text were derived by non-dimensionalizing equations 7 and 8 using the following dimensionless parameters.

$\tilde{V}_{cyt}=\frac{V_{cyt}}{\hat{V_{14}}}$ $\tilde{A}_{cyt}=\frac{A_{cyt}}{\hat{A_{14}}}$ $\gamma=-k_{b}C_{c}^{o}\bar{T}$ $\beta=k_{d}^{max}\bar{T}$ $\lambda_{d}= \frac{A_{nuc}^{14}\Gamma_{d}T}{V_{nuc}^{14}}$ $\lambda_{dc}= \frac{A_{nuc}^{14}\Gamma_{dc}T}{V_{nuc}^{14}}$

Thus, based on the two assumptions, the full six equation model was reduced to the two-equation model as shown in the main text.

# Effect of varying of varying the error criterion on analysis.

Changing the error cut-off value to 1.25 results in no significant change to the plots, while an error cut-off value of 0.75 results in no parameter set being selected as robust. However, a value of error equal to 1.0 results in the following changes (Fig. S5). For both 1x and 4x embryos, the length scale ratio (called $\rho$ in the main text) is always greater than 1, indicating that shuttling of Dl by Cact from dorsal to ventral regions becomes a necessary requirement (Fig. S5 A,B). The values of $\kappa$continue to be of order 1 or less. For 1x embryos, as the value of $\kappa$ decreases, there is an increasing spread in the values of amplitude ratios, while such a trend is not clear for 4x embryos (Fig. S5 C,D). Also, in all robust parameter sets we found that the Dl gradient decays to zero at the dorsal midline which is consistent with our deconvolution hypothesis.

# Approximate gradient width for dl 1x gradients

As the Dl gradient in embryos from mothers heterozygous for *dl* is not Gaussian-shaped, fitting it to a Gaussian gives an aberrant value for $\sigma$. To attempt to characterize the flat-topped gradients by a value of $\sigma$ equivalent to its closest approximation to a wildtype gradient, we did the following. First, by averaging ∼75 Dl gradients from 1x embryos, we created a “canonical” flat-topped gradient, normalized between zero and one, denoted $f_{50}\left( x \right)$. Next, we fit each 1x Dl gradient to this canonical gradient by allowing the spatial coordinate to be stretched (see Carrell et al., 2017; Liberman et al., 2009; Trisnadi et al., 2013 for examples). Therefore, for each 1x embryo $i$, we obtained a best-fit value of the spatial stretching factor, $\delta_{i}$.

Next, we calculated the area under the curve of a wt Gaussian:

| $I_{100}=\int_{0}^{1} \exp\left( -\frac{x^{2}}{2\sigma^{2}} \right)dx\approx\int_{0}^{\infty} \exp\left( -\frac{x^{2}}{2\sigma^{2}} \right)dx=\sigma\sqrt{2}\int_{0}^{\infty} \exp\left( -z^{2} \right)dz=\sigma\sqrt{\frac{\pi}{2}}$ | (10) |
| --- | --- |

where $z=x/\left( \sigma\sqrt{2} \right)$, and the change of the upper limit of integration to $\infty$ is valid because $\sigma\leq0.3$. The average width of the wildtype gradient is $\sigma_{wt}=0.152$, which implies $I_{100}=0.1880$.

Next, we calculated the area under the curve of $f_{50}\left( x \right)$, which was $I_{50}=0.2438$. Next, we computed the value of $\alpha_{50}$ makes $\alpha_{50}I_{50}=0.5I_{100}$, and found that $\alpha_{50}=0.3855$. Finally, to calculate the equivalent Gaussian-like width of the 1x Dl gradients, we computed the value of sigma that minimizes the following:

| $\varepsilon=\int_{x_{1}}^{x_{2}} \left[ f_{100}\left( x;\sigma\right)-\alpha_{50}f_{50}\left( x \right) \right]^{2}dx$ | (11) |
| --- | --- |

This value of $\sigma$, which we will call $\sigma_{1\times}^{eff}$ is 0.1283. In other words, if the average 1x embryo has 50% of the Dl in an average wildtype embryo, then the Dl gradient in an average 1x embryo looks most like a wildtype gradient with a width of 0.1283 (slightly narrower than the average wildtype gradient). Taking this base value of $\sigma_{1\times}^{eff}$, we can find the effective gradient width for each embryo $i$ by multiplying by $\delta_{i}$.

# Least squares calculations for thresholds and amplitudes in the empirical description

To estimate the necessary amplitude of the 1x and 4x canonical curves, with respect to wt, in order to achieve the observed gene expression (and given the observed shape and width of the Dl gradient), we constructed a least squares estimation. Let the objective function $f$ be the sum of the squares of error between the (empirical) Dl gradient at the locations of a given gene expression boundary and the estimated threshold for that gene:

| $f\left( \boldsymbol{\alpha},\boldsymbol{\theta},\boldsymbol{X,S} \right)=\sum_{g\in G} \sum_{\beta\in B} \left( \varepsilon_{\beta,g} \right)^{2}=\sum_{g\in G} \sum_{\beta\in B} \left( \frac{\alpha_{g}Dl_{g}\left( x_{\beta,g},\sigma_{g} \right)-\theta_{\beta}}{s_{\beta,g}} \right)^{2}$ | (12) |
| --- | --- |

…where the vector $\boldsymbol{\alpha}=\left[ \alpha_{1x},\alpha_{2x},\alpha_{4x} \right]$, the vector $\boldsymbol{\theta}=\left[ \theta_{sna},\theta_{sogv},\theta_{sogd} \right]$, the set of genotypes is $G=\left\{ \text{1x},\text{2x},\text{4x} \right\}$, the set of boundaries is $B=\left\{ sna,sogv,sogd \right\}$, and $x_{\beta,g}$ is the boundary location and $s_{\beta,g}$ is a measure of the variability for that genotype and boundary. In addition, the position array $\boldsymbol{X}$ and standard error array $\boldsymbol{S}$ are:

| $\boldsymbol{X}=\left[ \begin{matrix} x_{sna,1x} & x_{sna,2x} & x_{sna,4x} \\ x_{sogv,1x} & x_{sogv,2x} & x_{sogv,4x} \\ x_{sogd,1x} & x_{sogd,2x} & x_{sogd,4x} \end{matrix} \right]$ | (13) |
| --- | --- |

| $\boldsymbol{S}=\left[ \begin{matrix} s_{sna,1x} & s_{sna,2x} & s_{sna,4x} \\ s_{sogv,1x} & s_{sogv,2x} & s_{sogv,4x} \\ s_{sogd,1x} & s_{sogd,2x} & s_{sogd,4x} \end{matrix} \right]$ | (14) |
| --- | --- |

This can also be written more transparently as:

| $f\left( \boldsymbol{\alpha},\boldsymbol{\theta},data \right)=\sum_{\beta\in B} \left( \varepsilon_{\beta,1x} \right)^{2}+\left( \varepsilon_{\beta,2x} \right)^{2}+\left( \varepsilon_{\beta,4x} \right)^{2}$  $=\sum_{\beta\in B} \left( \frac{\alpha_{1x}Dl_{1x}\left( x_{\beta,1x},\sigma_{1x} \right)-\theta_{\beta}}{s_{\beta,1x}} \right)^{2}+\left( \frac{Dl_{wt}\left( x_{\beta,2x},\sigma_{2x} \right)-\theta_{\beta}}{s_{\beta,2x}} \right)^{2}+\left( \frac{\alpha_{4x}Dl_{wt}\left( x_{\beta,4x},\sigma_{4x} \right)-\theta_{\beta}}{s_{\beta,4x}} \right)^{2}$ | (15) |
| --- | --- |

This function can be minimized by linear least squares, with respect to varying $\alpha_{1x}, \alpha_{4x},\theta_{sna},\theta_{sogv},\theta_{sogd}$. The result of the optimum amplitudes and thresholds is given in Fig. 4D.

Additionally, this function can be minimized with respect to only $\theta_{sna},\theta_{sogv},\theta_{sogd}$ for any given set of $\left\{ \alpha_{1x},\alpha_{4x} \right\}$, which is plotted in Fig. 4D.

# Intensity calibrations for live images

As mentioned in the Methods Section of the main text, the laser intensity was measured each imaging session by collecting the 488 laser using the transmitted light channel with no sample to impede the light path. This measurement served as the laser calibration for all live embryos imaged during that imaging session. The GFP fluorescence in each live embryo was divided by the mean intensity of the corresponding laser calibration image, then multiplied by 1.75×10^4^, which was roughly the mean of all laser calibration images in the set of experiments. Multiplying by 1.75×10^4^ ensured Dl-GFP levels were maintained at roughly the same magnitude of intensity values as compared to before normalization by the mean of the laser calibration image.

As a second normalization step, the Dl-GFP intensities were normalized by the percent laser power.

In addition to using the laser calibrations, the fluorescence from Dl-GFP was also normalized by the H2A-RFP channel in two ways. First, there may be intensity variation along the DV axis due to uneven illumination. The uneven illumination could arise from a slight oblique angle between the z-axis of the light path and the z-axis (i.e., anterior-posterior axis) of the embryo. However, even if these axes are perfectly aligned, embryo geometry is not perfectly ellipsoidal, which implies the side with more curvature (the ventral side) may receive a higher level of illumination. To account for this, the intensities of the nuclei in the H2A-RFP channel were found, and for each time point, the Dl-GFP nuclear intensity was normalized by the H2A-RFP nuclear intensity. The resulting ratio of intensities was then multiplied by the median of the nuclear H2A-RFP intensities for that time point to maintain roughly the same magnitude of intensity values as compared to before normalization.

Second, the H2A-RFP channel was used to correct the entire time course for possible variations in distance from the embryo’s pole (i.e., z-depth). The image intensity will become weaker with increasing z-depth. Therefore, we normalized the entire Dl-GFP time series by a scalar corresponding to the median of all H2A-RFP nuclear intensities (for the entire timecourse) divided by the red laser power and divided by 100.

**Supplemental References**

1. O’Connell MD, Reeves GT. The presence of nuclear cactus in the early drosophila embryo may extend the dynamic range of the dorsal gradient. Baker RE, editor. PLoS Comput Biol. 2015;11: e1004159. doi:10.1371/journal.pcbi.1004159

2. Liberman LM, Reeves GT, Stathopoulos A. Quantitative imaging of the Dorsal nuclear gradient reveals limitations to threshold-dependent patterning in Drosophila. Proc Natl Acad Sci U S A. 2009;106: 22317–22. doi:10.1073/pnas.0906227106

3. Trisnadi N, Altinok A, Stathopoulos A, Reeves GT. Image analysis and empirical modeling of gene and protein expression. Methods. 2013;62: 68–78. doi:10.1016/J.YMETH.2012.09.016

4. Carrell SN, O’Connell MD, Jacobsen T, Pomeroy AE, Hayes SM, Reeves GT. A facilitated diffusion mechanism establishes the Drosophila Dorsal gradient. Development. 2017;144: 4450–4461. doi:10.1242/dev.155549


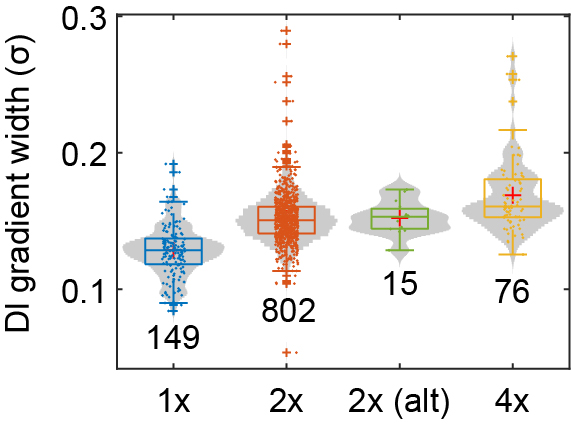
**Figure S1.** Box-and-violin plots of Dl gradient width measurements as depicted in Fig. 4B, with the additional alternate 2x *dl* fly line. The 2x alt fly line has one copy of endogenous maternal *dl* and one copy of the transgenic *dl* rescue construct (see Methods in main text).


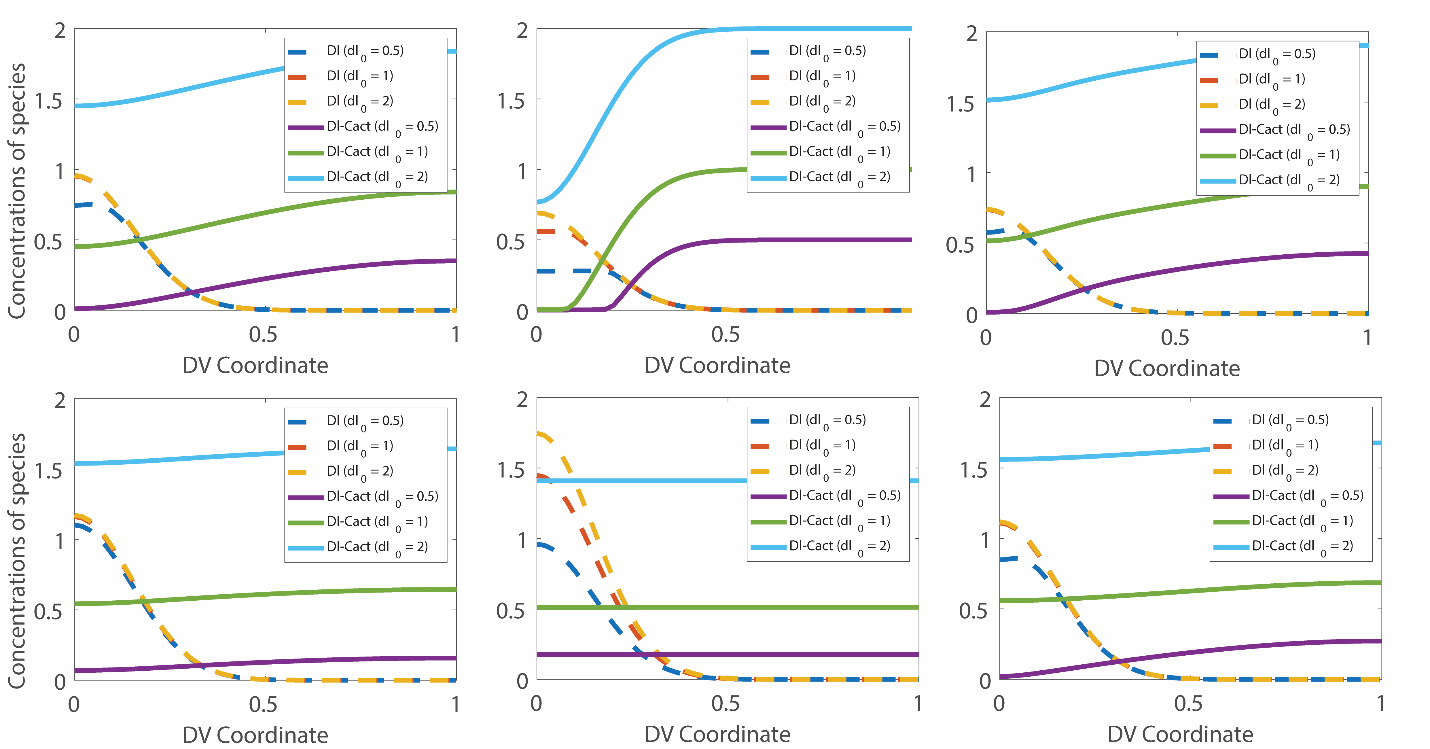


**Figure S2**. Concentration profiles of free Dl and Dl/Cact (robust parameters). This figure shows concentration profiles of free Dl & Dl/Cact, for parameter sets that were accepted as robust. The plots show non-zero concentration for Dl/Cact complexes at the dorsal midline at $x = 1$.


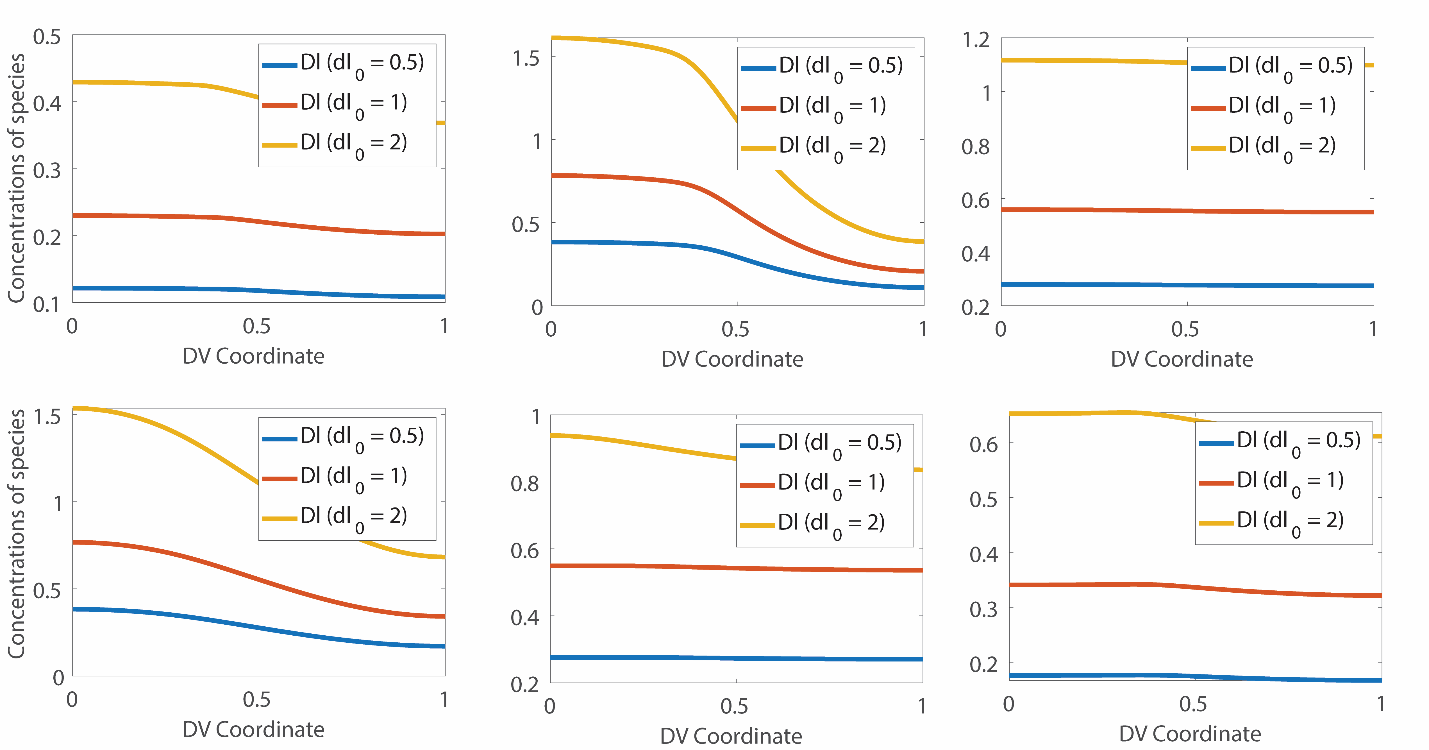
**Figure S3**. Concentration profiles of free Dl (non-robust parameters). This figure shows concentration profiles of free Dl, for parameter sets that were rejected as not robust. In most cases, concentration curves do not decay to zero at the dorsal midline.


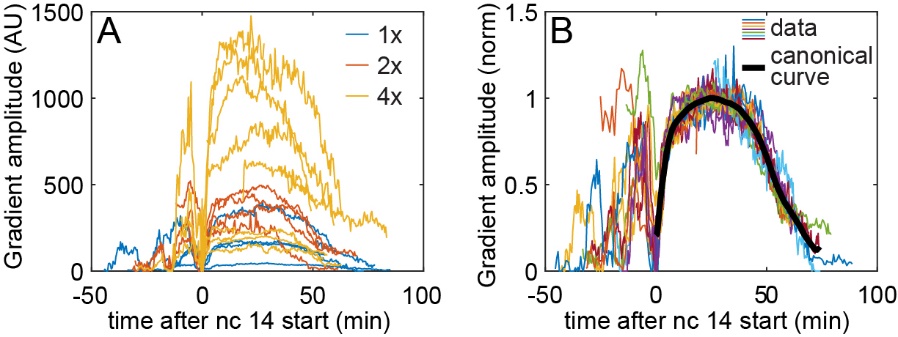


**Figure S4**. Live imaging data and creation of a canonical profile for $A(t)$. (A) Plot of each $A(t)$ obtained for each embryo, grouped by *dl-mgfp* dosage. (B) Plots of $A(t)$ from the same embryos in (A), but normalized so that their nc 14 interphases are all the same duration (roughly one hour) and their peak in nc 14 $A(t)$ is roughly 1. These nc 14 interphases are then averaged to obtain a canonical nc 14 $A(t)$ curve (black). See also Fig. 6C.

**Figure S5**. Distribution of robust parameter sets obtained for value of error equal to 1. (A) Plot of amplitude of ratio 1x/2x against $\rho$. (B) Plot of amplitude of ratio 4x/2x against $\rho$. (C) Plot of amplitude of ratio 1x/2x against $\kappa$. (D) Plot of amplitude of ratio 4x/2x against $\kappa$.

**D**

**B**


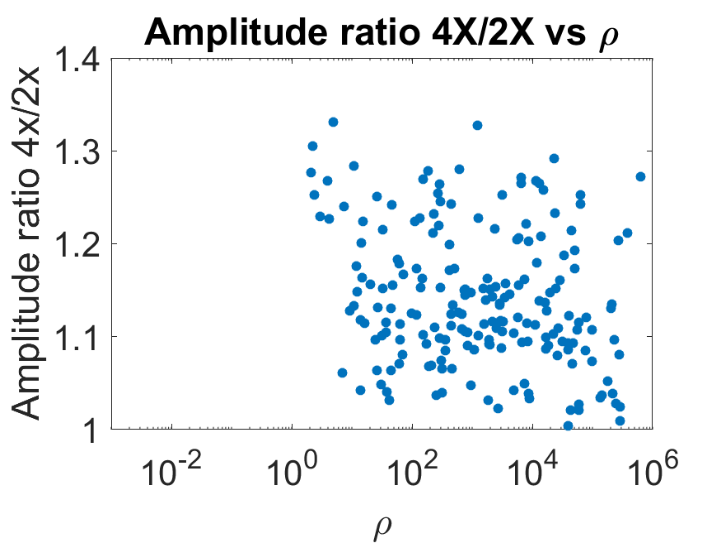

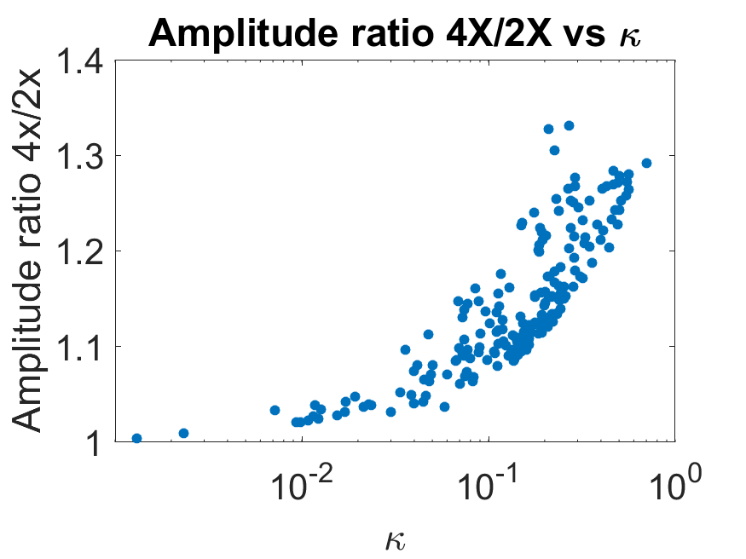

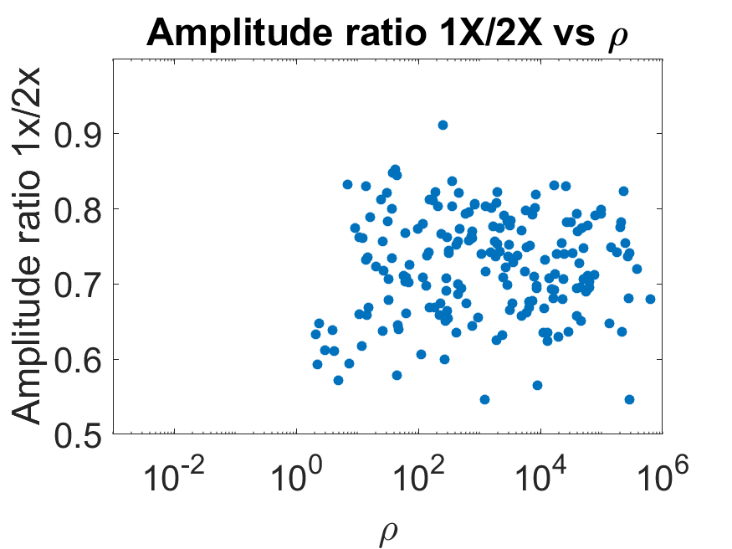

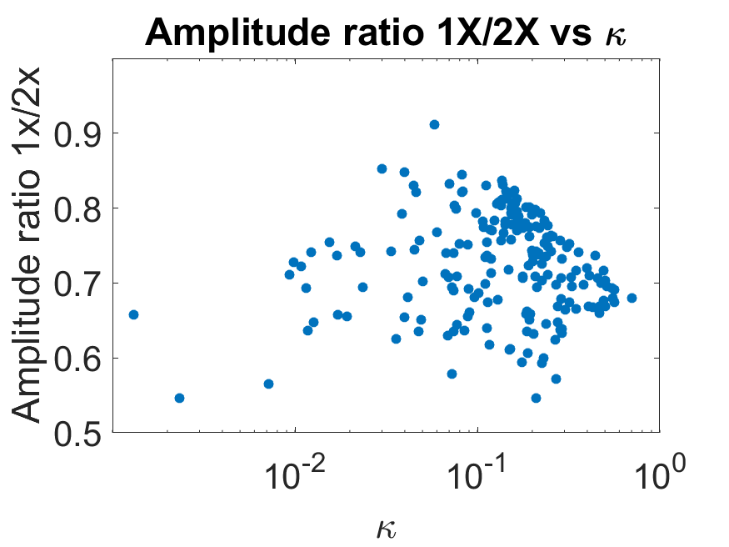


**A**

**C**
